# Towards annotating the plant epigenome: the Arabidopsis thaliana small RNA locus map

**DOI:** 10.1038/s41598-018-24515-8

**Published:** 2018-04-20

**Authors:** Thomas J. Hardcastle, Sebastian Y. Müller, David C. Baulcombe

**Affiliations:** Department of Plant Sciences, University of Cambridge, Downing Street, Cambridge, CB2 3EA United Kingdom

## Abstract

Based on 98 public and internal small RNA high throughput sequencing libraries, we mapped small RNAs to the genome of the model organism *Arabidopsis thaliana* and defined loci based on their expression using an empirical Bayesian approach. The resulting loci were subsequently classified based on their genetic and epigenetic context as well as their expression properties. We present the results of this classification, which broadly conforms to previously reported divisions between transcriptional and post-transcriptional gene silencing small RNAs, and to PolIV and PolV dependencies. However, we are able to demonstrate the existence of further subdivisions in the small RNA population of functional significance. Moreover, we present a framework for similar analyses of small RNA populations in all species.

## Introduction

Small RNAs (sRNAs) are approximately 20–30 nucleotide non-coding RNA molecules that play a key role in the regulation of genome function^1^. They are derived from long RNA precursors through the action of Dicer or Dicer-like ribonucleases^2^ and form effector ribonucleoprotein complexes with a member of the Argonaute superfamily^3^.

In plants, sRNAs are derived from thousands of genomic loci that fall broadly into two major categories^4^. In the first category, the precursor is transcribed by RNA polymerase IV (PolIV), converted to double-stranded RNA (dsRNA) through the action of the RNA dependent RNA polymerase RDR2, and processed by the Dicer-like DCL3 into 24 nt sRNAs. Canonically, these sRNAs will then operate within the RNA-directed DNA methylation (RdDM) pathway^5^. The majority of the targets of this pathway are transposons^6^, but there may also be indirect silencing effects on adjacent genes^7^.

The second major category of sRNA is transcribed *via* RNA polymerase II (PolII) into single-stranded RNA that forms a hairpin structure. The hairpin is then processed by various Dicer-like proteins, predominantly into 21 or 22 nt sRNAs. MicroRNAs (miRNAs), in which a single mature miRNA is produced in abundance^8^ are perhaps the best characterised subclass of hairpin-derived sRNAs. However, there are also many loci at which sRNAs are produced across the full length of the hairpin structure^4^.

Within these broad categories, there are several variations. A large subset of the PolIV-dependent sRNAs, for example, are also dependent on RNA polymerase V (PolV) that produces scaffold RNAs, allowing the recruitment of DRM2 and thus the initiation of *de novo* DNA methylation in the RdDM pathway^9^. There are also non-canonical RdDM loci in which PolII and the RNA dependent RNA polymerase RDR6 are required for the initiation of DNA methylation^5^.

Additional sRNA pathways are triggered by an sRNA, usually a miRNA, that targets a long single stranded RNA. This primary targeting event recruits an RNA dependent RNA polymerase (RDR), producing a dsRNA that is cleaved by DCLs into 21–22 nt sRNAs. These are known variously as tasiRNAs^10^, phasiRNAs^11^ or easiRNAs^12^, with the precise nomenclature dependent on the conditions required for biogenesis and their downstream effects. Further pathways for the production of sRNAs include natural-antisense siRNAs (natsiRNAs) produced from dsRNA formed from overlapping transcription events (cis-natsiRNA)^13^ or highly complementary transcripts (trans-natsiRNA)^14^.

The diversity of known mechanisms for sRNA biogenesis and their downstream functional behaviour suggests that multiple, perhaps interacting, classes of sRNA loci are to be found in plant species. In order to assess this hypothesis, we require a comprehensive map of sRNAs and sRNA loci. Provisional maps of sRNA loci exist^15–19^; however, these derive from very limited data sets and are based on heuristics that may not work for all classes of sRNA loci, datasets, tissues or conditions. In this manuscript we present a robust sRNA locus map for *Arabidopsis thaliana* that overcomes these limitations through the use of many replicated high-throughput sequencing libraries.

We use the empirical Bayesian approach implemented in the R package ${\mathtt{segmentSeq}}$^20^ to generate this map and annotate the sRNA loci with reference to internal properties of the loci and external data sets. Using a multiple correspondence analysis we identify nine classes of sRNA loci in the *A*. *thaliana* genome. Validation of this classification by comparison with external data supports the view that these nine classes represent features of the genome with biological significance.

## Results

**Figure 1:**
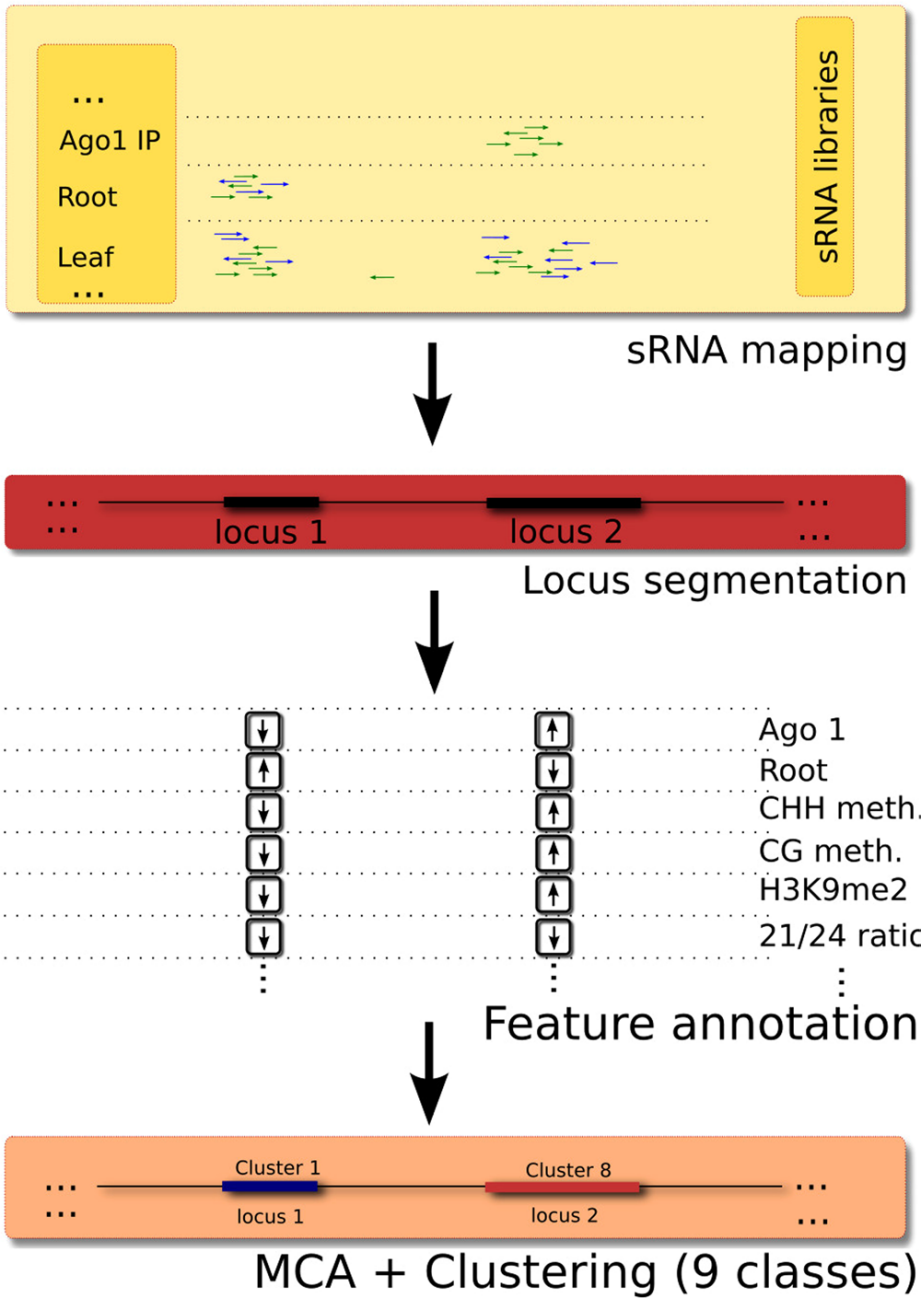
Summary of work-flow. Schematic diagram of the work-flow used to identify and classify sRNA loci. The yellow box depicts raw data (individual sRNAs are shown as green and blue arrows) used as a basis for segmentation (red box). Annotation features are determined from the loci and by comparison with external data sets and used to classify the loci (orange box).

Our approach to a classified sRNA locus map of *A*. *thaliana* (illustrated in Fig. 1) was to first identify genomic loci corresponding to sRNA reads in one or more of a large number of datasets. The data were derived from wild type tissues, mutant plants, and sRNA samples bound to different AGO proteins in immunoprecipitation (IP) experiments (Supplementary Table 1). We then annotated each locus based upon their characteristics (length, sequence composition, *et cetera*) and the sRNAs aligning to that locus (see Methods). We further annotated each locus with reference to external annotation sources describing genetic (e.g. genes and transposable elements) and epigenetic (e.g. histone modifications and cytosine methylation) features overlapping the loci. Finally, we annotated the loci based on external datasets describing the dependence of sRNA production on particular components of the sRNA pathways (e.g. PolV and PolIV).

From the initial inspection of these loci it was clear that they could not be classified by simple correspondence with annotation features either singly or in simple combination. We therefore applied a multiple correspondence analysis based on the annotation data, reserving certain classes of annotation data for validation.

### Segmentation mapping of sRNA loci

Our sRNA locus map is based on 98 sRNA libraries encompassing 54 replicate groups (Supplementary Table 1). After initial trimming and filtering steps (see Methods) which also excluded known miRNA sequences, the libraries contained a total of 16.2 million non-redundant (175 million redundant) sRNA reads mapping to the *Arabidopsis thaliana* genome. The libraries varied widely in the total number of sequenced sRNAs (see Supplementary Table 1). Library scaling factors (a surrogate for library size) were calculated as in Hardcastle^20^.

In order to identify small RNA loci, we employed the empirical Bayesian methods implemented in the Bioconductor package ${\mathtt{segmentSeq}}$^20^. This method uses the number of sequenced sRNAs at each potential locus in each sample to compute posterior likelihoods that the region is an expressed sRNA locus within each replicate group. For annotation and classification analysis we selected loci with a false discovery rate of less than 5% in at least one replicate group and the final outcome was a map of 24559 loci (available in Supplementary Materials). Assuming independence of the likelihood of expression of each loci over replicate groups, we estimate that 465.6 (1.89%) of these loci are false positives (i.e., there is no true sRNA expression in these loci).

**Figure 2:**
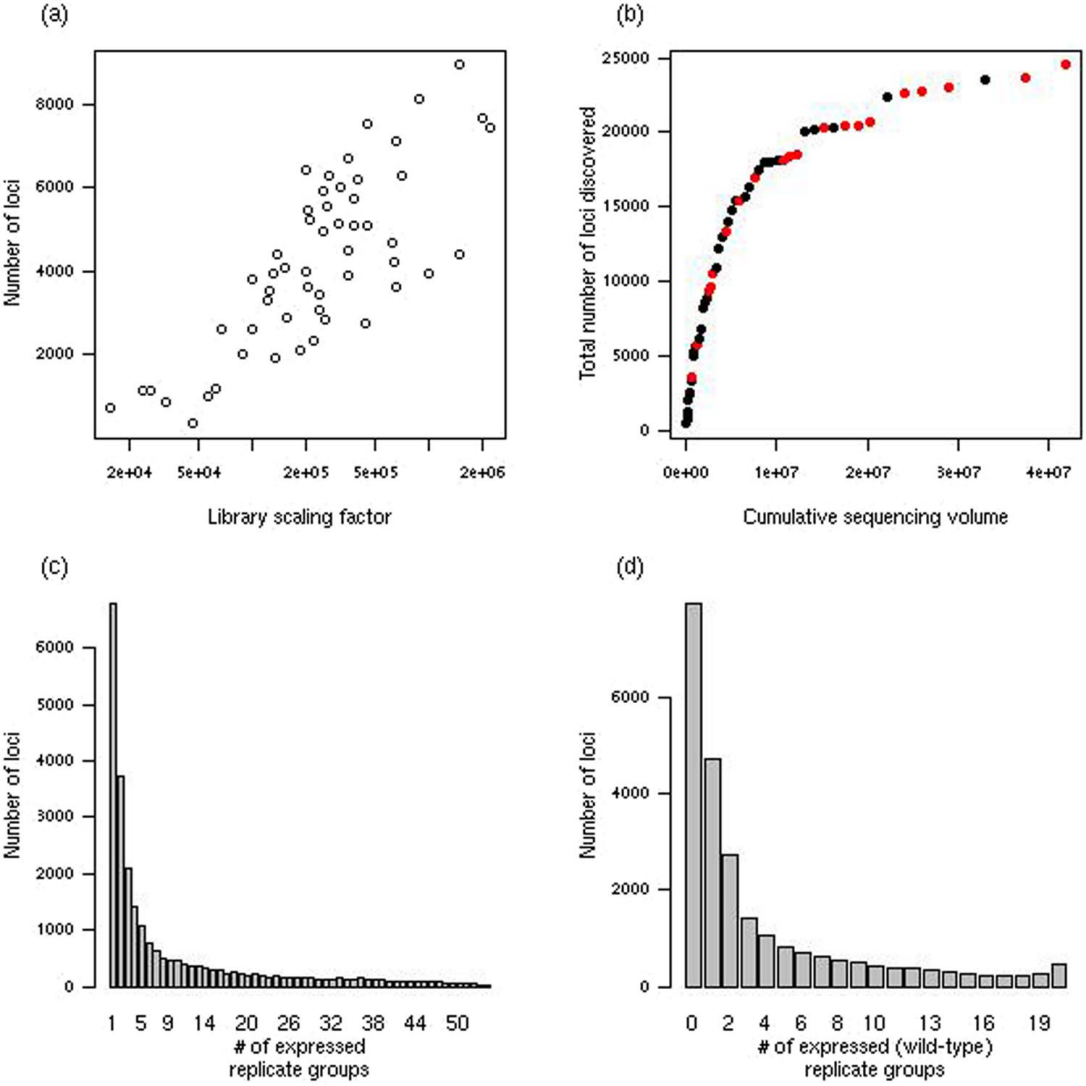
Locus discovery. The number of loci discovered varies substantially between replicate groups and is heavily dependent on sequencing depth. The number of loci identified in individual replicate groups against average library scaling factor is shown in (**a**), while the number of loci identified by cumulative sequencing volume for each additional replicate group (sorted by average library scaling factor) is shown in (**b**). The frequency of the number of replicate groups in which loci are expressed is shown in (**c**). The frequency of the number of wild-type replicate groups in which loci are expressed is shown in (**d**).

The 24559 identified loci cover 8.9 MB (7.47%) of the *Arabidopsis thaliana* genome and they vary in size from single stacks of 15 nt reads to a locus 15181 nt in length, with a median length of 132 nt. The number of loci within a replicate group correlates strongly with the average sequencing depth (Fig. 2(a)) suggesting that replicate groups with low average sequencing depth are likely to under-represent the number of loci expressed. From the cumulative sequencing volume of the combined datasets, however, the total number of loci is approaching saturation (Fig. 2(b)), suggesting that the majority of small RNA loci present in the Col-0 strain of *Arabidopsis thaliana* have been identified within these data.

We defined an identified locus as expressed within a particular replicate group if the posterior likelihood of expression exceeded 50% (i.e., the locus is more likely expressed than not within the replicate group). On this criterion, 6801 (27.7%) loci are expressed in only one replicate group, while only 18 (0.07%) loci are found in all 54 replicate groups (Fig. 2(c)) This pattern is likely due, in part, to the low depth of many sequencing libraries and to the use of knockout mutant libraries in which many sRNA loci will not be expressed. For the wild-type samples there were 16580 loci of which 4717 (28.4%) are expressed in a single wild-type sample, and 462 (2.79%) are expressed in all samples. For the wild-type samples, the distribution is bimodal (Fig. 2(d)), suggesting that there may be a meaningful distinction between ubiquitously expressed sRNA loci and those that are specific to some experimental condition.

### Annotation of sRNA loci

Annotation of the loci was based on both characteristics intrinsic to the sRNA loci, and those obtained through comparison to existing annotations and other sequencing data. The intrinsic characteristics derived from sRNA datasets were based on the strand bias of the sRNAs, the abundance ratio of 24 nt to 21 nt sRNAs, the length of the locus, the predominant 5′ nucleotide and phasing (see Table 1 for a full list of the features that are included). Extrinsic features were derived from external ChIP-Seq or BS-Seq libraries as well as annotations including different types of transposon, types of repeated sequence (direct, dispersed, inverted), and other genetic elements from The Arabidopsis Information Resource (TAIR10)^21^. We also included natural antisense transcripts(NATs)^22^, long intergenic non-coding RNAs (lincRNAs)^23^ and inverted repeats (see Methods) as additional annotation characteristics because they have been associated with sRNAs.

**Table 1:** Feature statistics.

Feature Type	Categories			
Loci width	(0, 50]	(50, 2e + 03]	(2e + 03, Inf]	
	8589	15282	688	
Expression	yes	no		
	20896	3663		
Phasing	none	moderate	high	
	20250	2176	2133	
21/24 ratio	low*	moderate	high	unknown
	168	13740	3071	7580
Tissue	specific	intermediate	common	
	12684	8844	3031	
Repetitiveness	low*	moderate	high	unknown
	5199	2569	9025	7766
Strand ratio	strong	medium	no*	unknown
	7596	4771	3956	8236
DCL2/3/4 dependent	yes	no		
	6868	17691		
RDR2	indep.	unknown	dependent	
	1852	9189	13518	
Pol IV	indep.	uknown	dependent	
	2877	10209	11473	
Pol V	indep.	not known	dependent	
	5877	11364	7318	
CpG	low*	moderate	high	unknown
	4103	2108	9694	8654
CHG	low*	high	unknown	
	6264	10142	8153	
CHH	low*	high	unknown	
	11153	9987	3419	
H3	none*	low	moderate	high
	6178	14519	2553	1309
H3K9me2	low*	moderate	high	unknown
	11258	4984	88	8229
H3K27me1	low*	moderate	high	unknown
	6599	10044	672	7244
H3K27me3	low*	moderate	high	unknown
	635	6400	6244	11280
H3K36me2	low*	moderate	high	unknown
	3364	9559	3245	8391
H3K36me3	low*	moderate	high	unknown
	2635	8586	4437	8901
H3K4me2	low*	moderate	high	unknown
	2269	6555	3637	12098
H3K4me3	low*	moderate	high	unknown
	2798	7200	3106	11455
H3K9Ac	low*	high		
	23047	1512		

Each locus was assigned to one of the categories of each feature (see Methods for details). Default categories (see Methods) are indicated where relevant by the *symbol. 5′ nucleotide statistics were excluded from this table due to the large numbers of these categories.

### Multiple Correspondence Analysis

Many of the locus annotation data are categorical in nature; for example, a given locus either overlaps a transposable element or it does not. Others are effectively continuous over a wide range; for example, the locus length. However, we find that those features which are continuous in general derive from a multimodal distribution on which a natural classification can be imposed (see Supplementary Figures 8–22). To facilitate interpretation and analysis we developed methods to bin the continuous data into categories defined by the data (see Methods). We annotated the loci with respect to these categories as defined in Table 1.

We were then able to apply a multiple correspondence analysis to the annotated loci. We used the R package ${\mathtt{FactoMineR}}$^24^ to perform MCA with individual loci as objects and including all features described in Table 1 as covariates. Genome annotation was excluded from the covariate list to serve as a standard with which to evaluate clustering efficiency.

**Figure 3:**
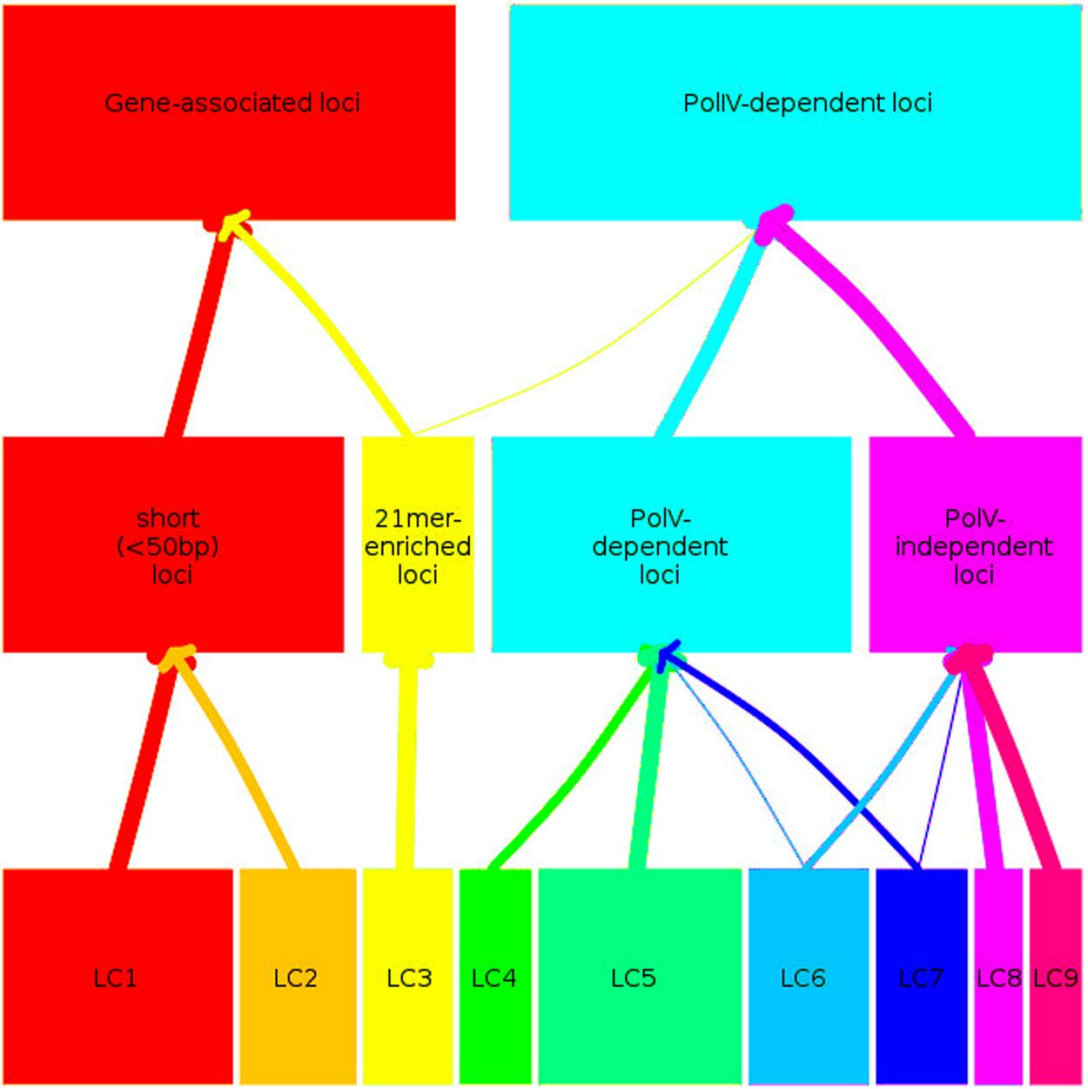
Class hierarchy. Structure of classes for *k* = 9, 4 and 2. Individual locus classes are identified as LC1, LC2 *et cetera*, while larger clusters are described by dominant biological features. The thickness of the arrows indicates the proportion of data in clusters created with higher *k* that are present within the clusters found in lower *k*.

Following dimension reduction through MCA (see Methods), K-means clustering was performed to establish classes of sRNA loci. Appropriate values for the numbers of clusters and the level of dimension reduction were determined (see Methods for more details) by examining the additional percentage of variance explained with the inclusion of each additional dimension, and by performing stability analyses by subsampling the loci (Supplementary Figure 7A,B). These analyses suggested that the first six dimensions of the MCA analysis could provide stable and informative data. To identify the number of clusters present in the data, we again examined the stability of the results under a subsampling procedure, the gap statistic of Tibshirani^25^ and the normalised mutual information between the identified clusters with annotated feature overlap (Supplementary Figure 7C,D). Taken together, these measures suggest that two primary categorisations of sRNA loci are to be found in *Arabidopsis thaliana*. Each of these can be split into two secondary categorisations, which themselves can be further subdivided, resulting in nine tertiary classes of sRNA loci. (Fig. 3).

#### Feature associations

**Figure 4:**
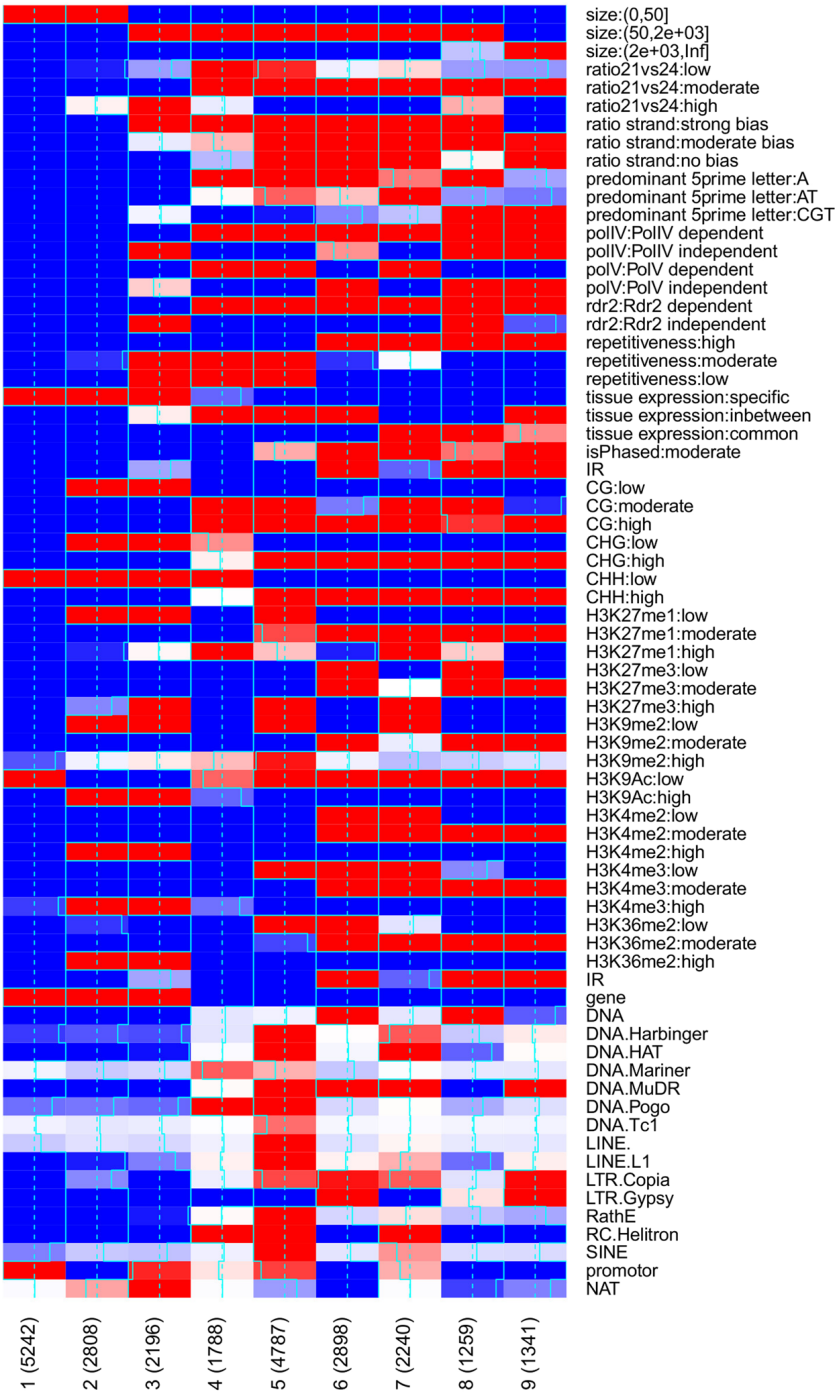
Feature associations. Enrichment of association for selected features in each locus class. Red indicates positive and blue negative association with the intensity of the colour measuring the level of significance. Numbers in brackets indicate the number of loci identified in each locus class.

Having established a set of nine locus classes (LC1-9), we next characterised them by computing relative enrichment for the loci features listed in Table 1 within each respective cluster. Figure 4 shows the significance of enrichment or depletion for selected features in the nine classes. Supplementary Figure 4 shows significance of enrichment or depletion for all features included in the MCA with significant association.

Apart from gaining insights into the biogenesis and functional roles of the found LCs, the obtained associations can also be used to infer a hierarchical relationship between the locus classes whilst simultaneously identifying the features responsible for distinguishing between them. The primary division between locus classes (LC) is that between LC1-3 and LC4-9.

The set of small RNA loci identified within LC1-3 is unique in significantly associating with genes, with 79.2% of the loci overlapping genes falling in one of the three locus classes. These loci also significantly associate with loci expressed non-ubiquitously, containing 65.5% of all loci with tissue-specific expression (see Methods for a definition of tissue specificity). These classes show significant anti-association with cytosine methylation, and together contain 68.4% of all loci with low CHH context methylation.

Locus classes LC4-9 are defined by their significant association with epigenetically activated small interfering RNAs (easiRNAs)^12^, and with the PolIV and RDR2 pathways. Together, these locus classes contain 92.6% of easiRNA, 97.7% of PolIV-dependent, and 96% of RDR2-dependent loci. These classes of loci also significantly associate with cytosine methylation, containing 97.1%, 99.3% and 99% of loci identified as showing high CG, CHG and CHH context methylation respectively.

#### Gene-associated sRNA loci

The set of gene-associated sRNA loci (LC1-3) can be further partitioned. LC1 and LC2 appear more closely related, both strongly associating with the smallest class of small RNA loci. 87% of small RNA loci shorter than 50 nt in length are in LC1 and LC2. These classes differentiate in their overlap with gene and promoter regions. 77% of the loci in LC2 overlap with a gene region, while only 38% of the LC1 loci show similar overlap. However, 23% of the LC1 loci overlap with promoter regions (defined as the 500 nt upstream of gene start sites), the strongest level of association with promoters of any locus class. This is substantially different to the behaviour of LC2, which shows significant depletion of overlap with promoter regions. LC2, in contrast to LC1, significantly associates with high levels of H3K4me2, H3K4me3, H3K36me2 and H3K9Ac, histone marks typically associated with mRNA activation^26^, and with genes observed to be expressed in Luo *et al*.^22^ (Supplementary Materials).

We hypothesize that LC1-2 may consist not of true sRNA biogenesis loci but target sites for sRNAs acting in trans, particularly at the gene regulatory level. Analysis of the uniqueness of sequences within each locus class offers limited support for this; 67% and 59% of the sRNA sequences found in LC1 and LC2 are also present in loci belonging to locus classes LC3-LC9. However, this leaves several thousand sequences which only appear in LC1 and LC2.

The majority of LC3 class loci are longer than those in LC1-2; 73% of LC3 loci are greater than 50 nt in length. LC3 appears to contain miRNA-like sRNA; 89% of LC3 loci are highly enriched for 20-21mers and this class contains 63% of all such loci. 63% of the LC3 loci show strong strand bias and 51% of the LC3 loci are identified as PolIV-independent loci with only 7% associated with PolIV-dependence. LC3 is also strongly enriched for loci containing small RNAs having a cytosine at their 5′ base, containing 35% of all such loci. LC3 shows a similar enrichment to LC2 for high levels of H3K4me2, H3K4me3, H3K36me2 and H3K9Ac, as well as with genes observed to be expressed in Luo *et al*.^22^.

#### Methylome-associated loci

Locus classes LC4-9, associated with cytosine methylation and PolIV and RDR2 dependence, can be partitioned into two major subclasses. The first consists of the primarily PolV-dependent classes LC4,5&7, the second, of the PolV-independent classes LC6,8&9. Classes LC4,5&7 together contain 86% of the loci identified as PolV-dependent; conversely, LC6,8&9 contain 73% of the loci identified as PolV-independent. This is the standard division of PolIV-dependent sRNA loci^27^ and that implied by a kmeans clustering at *k* = 4 (Fig. 3). However, multiple feature classifications cross this division, implying that several competing influences may operate on these sRNA loci.

#### PolV-dependent loci

Features distinctive to the PolV-dependent locus classes are promoter overlap, for which LC4,5&7 are enriched (LC4 non-significantly) and LC6,8&9 are significantly depleted, and an overlap with loci annotated as mobile in shoot-to-root grafts^28,29^, containing 84% of all such loci. The LC4,5 & 7 grouping is also unique in that all three locus classes are enriched for low numbers of 20–21 nt sRNAs compared to 23–24 nt sRNAs, containing 89% of all such loci. Although the transposable element superfamilies overlapped by these locus classes vary, all three are enriched for overlap with RC/Helitron elements, together containing 78% of all loci overlapping this superfamily.

Within the class of PolV-dependent loci, there exist significant distinguishing features that separate the three locus classes LC4,5&7. LC4 is unique in containing a significant proportion of loci with low CHG (30% of LC4 loci) and CHH (55% of LC4 loci) context methylation, and in lacking any enrichment for moderate or high phasing. LC5 & LC7 are unique amongst the PolV-dependent loci classes in showing enrichment for high levels of the histone marker H3K27me3 (associated with expression inactivation), in common with LC3.

#### PolV-independent loci

The PolV-independent locus classes LC6,8&9 are unique in their enrichment for loci overlapping with inverted repeat regions, together containing 73% of such loci, and in their association with median to low levels of H3K27me3 (60% of loci with median levels and 98% of loci with low levels of H3K27me3) and with median levels of H3K9me2 (83% of such loci). In general these locus classes are also associated with loci having median (93% of such loci) or high (93% of such loci) levels of histone accumulation. The PolV-independent classes are significantly enriched for PolIV-dependence. However, unlike the PolV-dependent classes they also show (along with LC3) significant enrichment for PolIV-independent loci. These classes are all significantly depleted for overlap with RC/Helitron elements, and all show enrichment for Gypsy elements (LC8 non-significantly), together containing 85% of all loci overlapping these elements. LC6 and LC8 are unique in their enrichment for overlap with DNA elements, containing 64% of loci overlapping these. Within the PolV-independent locus classes, LC8 and LC9 are notable in containing 70% of the loci significantly depleted in sRNAs with adenine as the 5′ base.

LC8 is also distinguished amongst the PolIV-dependent locus classes in containing significant numbers of RDR2-independent loci (31% of the LC8 loci are identified as RDR2-independent), of loci with a high ratio of 21/22 to 23/24 sRNAs (16% of the LC8 loci), and loci associated with mRNA expression (28% of the LC8 loci). LC9 is unique in containing 94% of the loci greater than 2000 bases in length, with such loci making up 48% of the class. It is also notable in a significant lack of loci exhibiting strong strand bias; only 2% of the LC9 loci show strong strand effects compared to 31% globally.

#### Feature associations independent of PolV dependency

LC7 shares several features in common with LC8 and LC9, despite the difference in PolV dependency. Together, these locus classes contain 91% of all sRNA loci exhibiting strong evidence of phasing; 46% of these loci are found in LC9 alone. These three classes also contain 84% of the loci commonly expressed across samples, and are all significantly enriched for DCL2,3,4-independent loci, together containing 45% of such loci. With the addition of LC6, this set of locus classes all exhibit high repetitiveness, with between 80% and 98% of all loci in these classes being classified as highly repetitive, compared to a global average of 37%. LC6-9 also contain between 69% and 75% of the loci overlapping moderate levels of histone marks H3K4me2, H3K4me3, and K3K36me2, and so may have some association with mRNA activation.

### Locus class validation

To evaluate the classes identified by multiple correspondence analysis we consider the association between the locus classes and several features not used to generate the classification.

#### Genomic location

**Figure 5:**
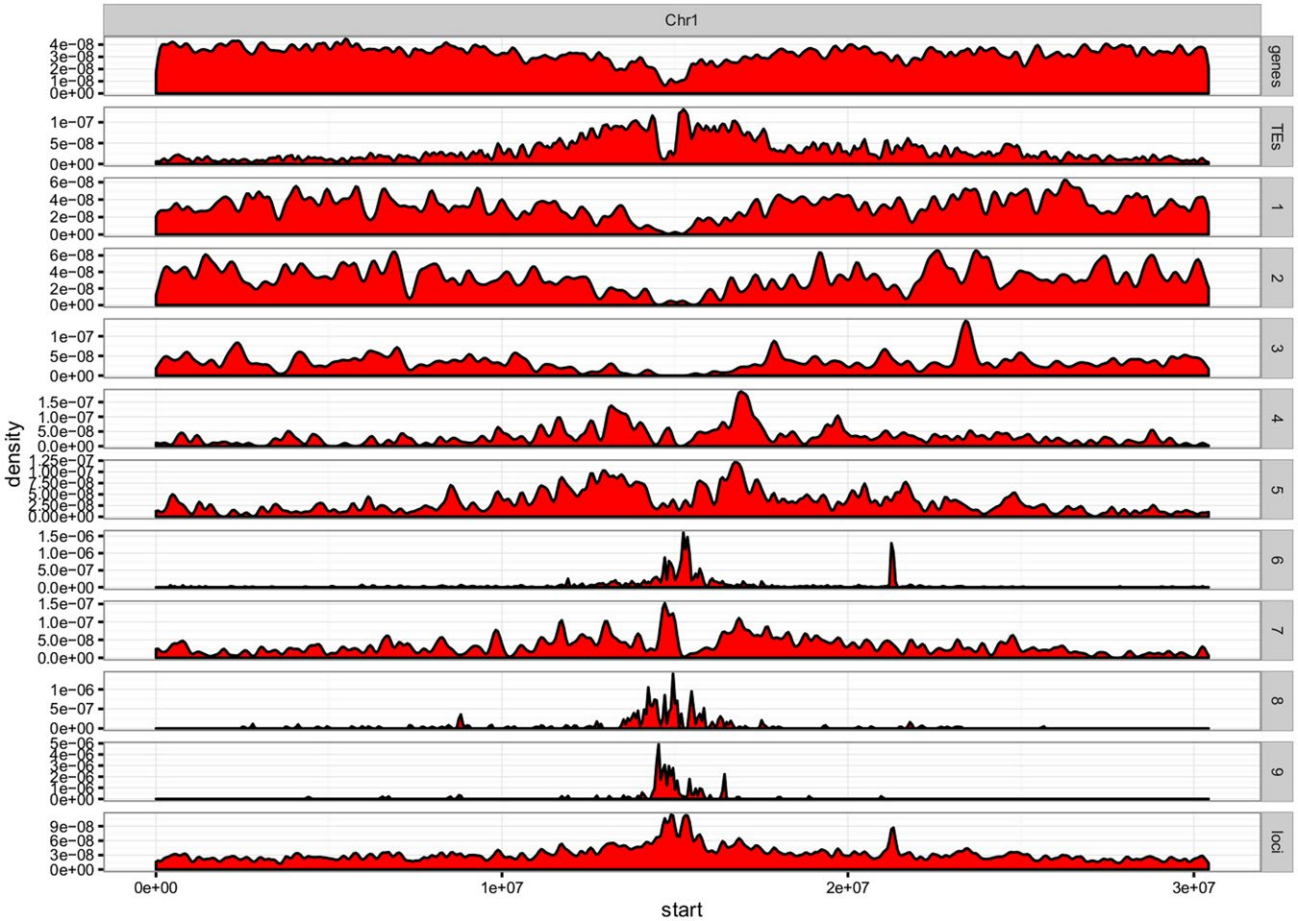
Genome wide sRNA class profiles. Genome wide density of small loci for all 9 loci classes (panels 3 to 11) were plotted along the *Arabidopsis thaliana* chromosome 1 as well as total loci density (last panel), transposon density (panel 1) and gene density (panel 2) for comparison.

The genomic locations of the loci within the locus classes (Fig. 5) reflects the broad partition between LC1-3, LC4,5&7 and LC6,8&9. LC1-3 are found predominantly in chromosomal arms. LC4,5&7 are predominantly peri-centromeric, and LC6,8&9 are predominantly centromeric (see Supplementary Figure 5 for coverage of the entire genome).

#### Cytosine methylation pathways

**Figure 6:**
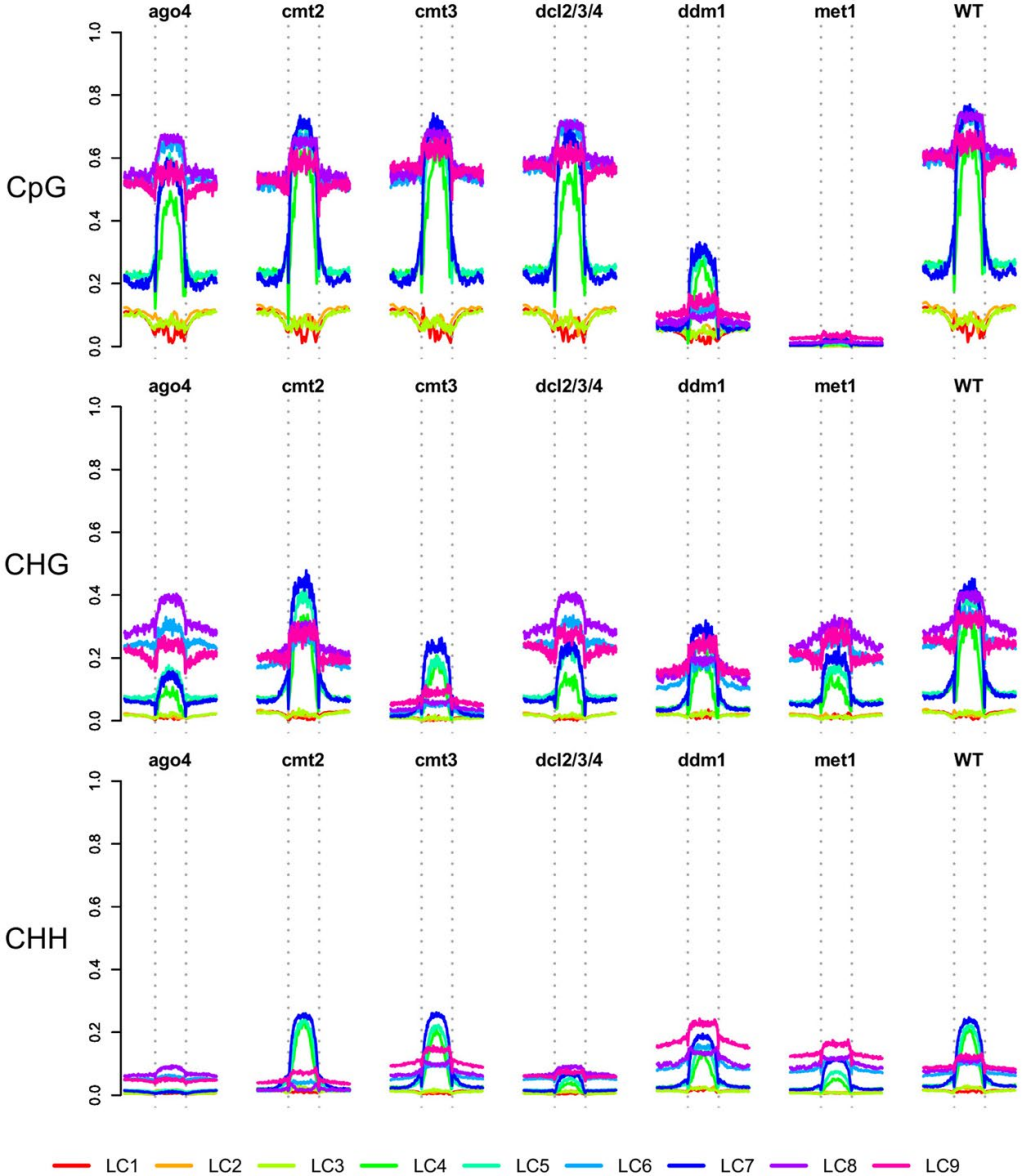
Locus class profiles of methylation. The average methylation level in CpG, CHG and CHH context is shown for each locus class in a selection of mutants and wild-type data.

Profiles of methylation across the sRNA loci contained within each locus class, and the variation in these profiles in different mutants of the methylation pathways also support the hierarchical classification we propose here. Figure 6 shows the average level of methylation across sRNA loci for each locus class for the three major methylation contexts in a variety of mutants^30^. The gene-associated sRNA locus classes LC1-3 show no methylation in any context, and a dip in methylation in the CpG context relative to background levels. The PolV-dependent locus classes, LC4,5&7 lie within regions of comparatively low background methylation, but have high methylation in all three contexts in wild type samples. LC4 loci show on average slightly lower methylation than LC5 & 7 loci. The PolV-independent locus classes lie within regions of high background methylation, but show a sharply defined increase in methylation at the locus boundaries. There is some evidence of a dip in methylation immediately outside the methylation for the LC9 loci, which also show a slightly lower average methylation than LC6 & LC8 methylation in wild-type samples in CpG and CHG contexts.

The loss of methylation in the *ago4* and *dcl2/3/4* mutants in the CHG and CHH contexts demonstrates the dependence of methylation in the PolV-dependent locus classes on the RdDM pathway^5^. Conversely, CHG and CHH context methylation in the PolV-independent locus classes is clearly driven predominantly by the CMT3 and CMT2 pathways^30^ respectively and is largely independent of the RdDM pathway. Mutation of the chromatin remodeling protein DDM1^31^ induces the largest variation in effects between the locus classes, reducing CpG methylation in the PolV-independent locus classes substantially with a lesser effect on the PolV-dependent locus classes. In the CHH context, it is notable that methylation in LC9 alone increases in the *ddm* mutant relative to wild-type levels. In the *met1* mutant, CHG and CHH context methylation is reduced in the PolV-dependent locus classes. However, methylation in the PolV-independent classes is generally unchanged, except for an increase in CHH context methylation in LC9 and a reduction in methylation in the CHG context in the LC8 loci. It is also perhaps notable that the sharp change in CHG context methylation at the locus boundaries is lost for LC8, but for no other locus class.

#### Length distributions

**Figure 7:**
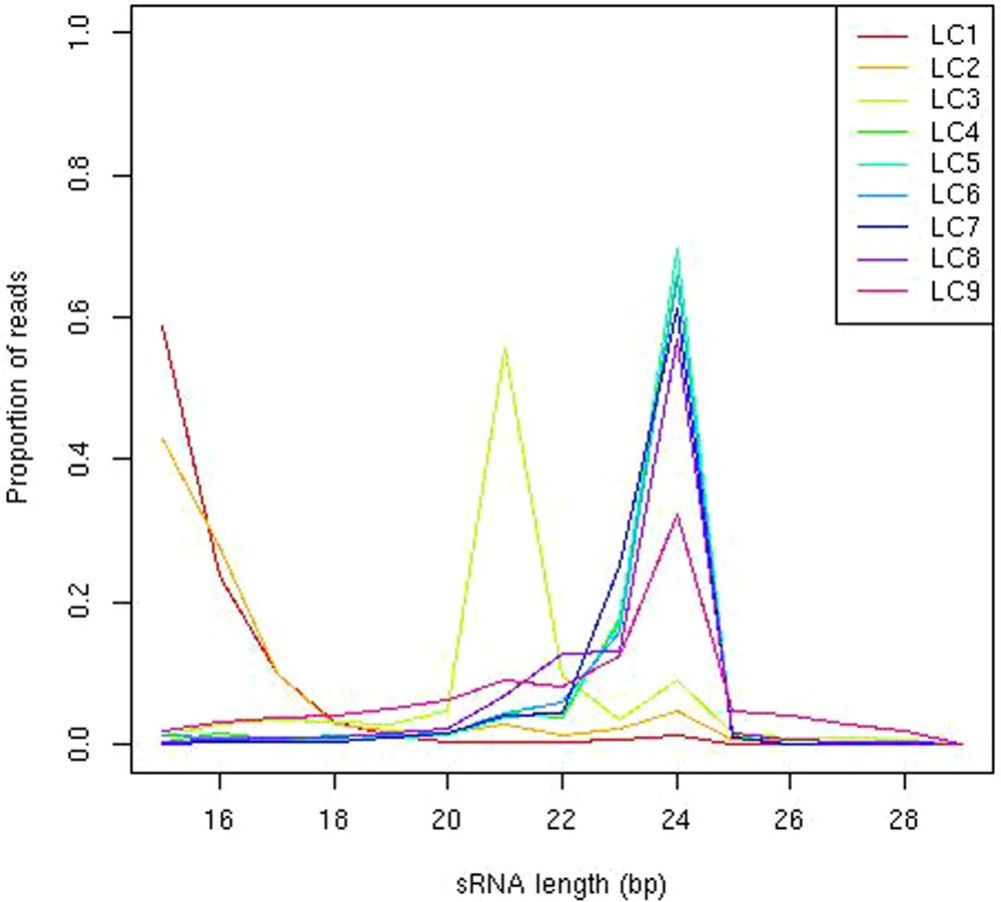
Distributions of small RNA lengths. Lengths of small RNAs segregate with respect to locus class.

The length distributions of small RNAs within each locus class (Fig. 7) are also informative. These emphasise the distinction between LC1&2 and LC3, with LC1&2 being strongly enriched for reads shorter than 18 nt in length while LC3 predominantly consists of 21mers. LC9 also distinguishes itself from the other PolIV-dependent locus classes in containing a wider variety of sRNA lengths.

#### Cluster representatives

**Figure 8:**
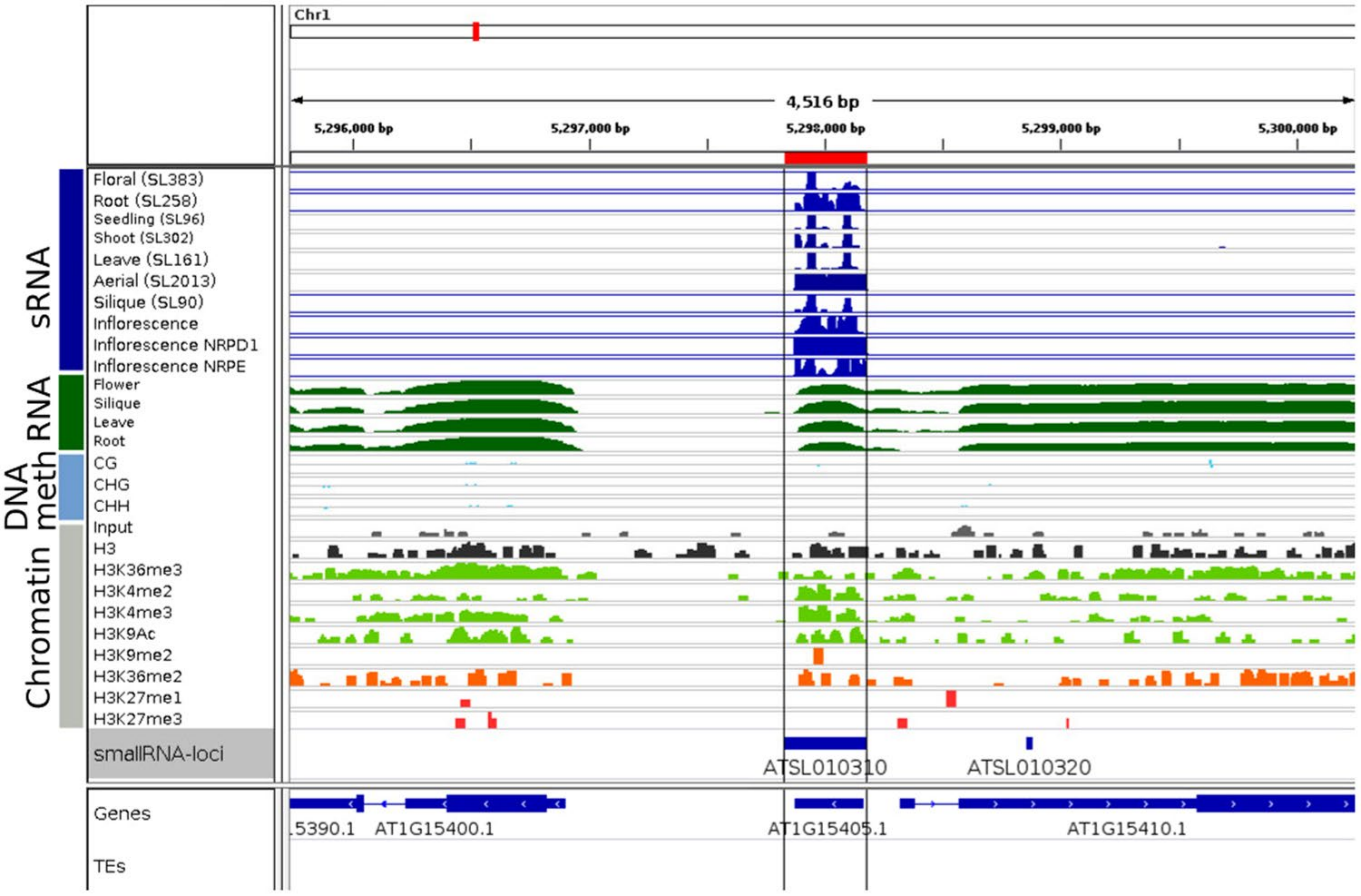
Genome browser view of the LC3 paragon. Top tracks (blue) depict sRNA coverage for a subset of libraries used in this study. The green tracks below show wild-type RNA-Seq libraries for various tissues obtained from Liu *et al*.^23^ which is followed by wild-type DNA-methylation tracks for CG, CHH and CHG contexts obtained from Stroud *et al*.^30^. Histone marks shown further below are indicated with a grey box and subdivided into green and red tracks reflecting their repressive associations with red indicating repressive and green active marks. Data were obtained from Liu *et al*.^23^. sRNA loci are shown on the track below with individual loci shown as blue bars.

Having described locus class characteristics in general we next set out to find loci serving as representative examples for a visual assessment and better understanding of the locus classes. To find the most appropriate representative loci for each locus class we used the concept of paragons as implemented in ${\mathtt{FactoMineR}}$ which identifies a set of loci close to the respective locus class center. Supplementary Figure 6 shows an example paragon for each LC. For example, the 352 nt long locus ATSL013100 (see Fig. 8) was identified as the paragon for LC3. In line with other LC3 characteristics, this loci shows sRNA expression in all tested tissues, has no preferential 5′starting letter, exhibits strong strand specificity, has a low repetitiveness score and produces mostly 21 nt sRNAs. This locus is known to produce long RNA in various tissues, which likely serve as the precursor to sRNA production. The chromatin state also conforms with long RNA expression, showing enrichment with activating histone marks including H3K4me2/3,H3K36me3 as well as H3K9ac, but not with repressive marks such as H3K9me2 and H3K27me1. Furthermore, DNA methylation is absent in all contexts and mutant data also suggest independence of PolIV, RDR2 as well as PolV making it likely to be involved in PTGS as opposed to TGS (such as RdDM). The paragon for LC2 (ATSL242410) indicates a similar epigenetic state, however it also shows high tissue specificity and is rather short, spanning only 25 nt, indicating a production of only one primary sRNA species.

In contrast, LC5 (ATSL000480) paragon embodies a classic PolIV dependent loci, losing sRNA production in the PolIV and RDR2 mutant, but also in the PolV mutant. The locus has a length of approximately 2 kb, is enriched in 24 nt sRNAs and shows methylation in all contexts. The LC4 paragon (ATSL079310), has a low repetitiveness score (0.07) but, in contrast to LC3 almost exclusively produces 24 nt sRNAs. It is also highly strand specific and tissue specific and overlaps an RC/Helitron type transposon; this superfamily of transposable elements is highly enriched in LC4 (see Fig. 4). The LC7 paragon ATSL172700 also overlaps with an RC/Helitron transposable element and shows high methylation in all contexts but is positioned in a H3 free region as opposed to the LC8 paragon ATSL274530. Finally, the LC9 paragon ATSL095900 is positioned in amongst a cluster of long LC9 loci with strong enrichment for 24 nt siRNAs.

We are also able to find additional sRNA loci closely related to well-studied sRNA producing regions. IR71 and IR2039 were identified as corresponding to sRNA loci ATSL133150 and ATSL172610 respectively. Both these loci are enriched in 21 nt sRNAs, being hierarchically processed first by DCL2 and DCL3, then secondarily by DCL4^32^. Additionally, both exhibit high DNA methylation in all sequence contexts and are members of LC9 which is found to be enriched in inverted repeats (see Fig. 4).

## Discussion

We have generated a detailed map of sRNA (precursor) loci in Arabidopsis based on large volumes of sequencing data. We identified a number of discriminatory factors between these sRNA loci and combined these with histone modification, methylation and annotation data. Based on an MCA analysis of these factors, we identify nine distinct classes of sRNA loci based on functional pathways, association with histone markers, *et cetera*. We show these different classes to have functional significance.

Figure 3 shows that the expected^27^ subdivisions of PolIV-dependent and PolIV-independent loci are found within these data. A further subdivision of the PolIV-dependent loci into PolV-dependent and PolV-independent classes also holds for these data. However, the further subdivisions identified within these major classes suggest a greater diversity of sRNA classes than can be explained by the operation of these polymerases alone. For example, the PolV-independent locus classes LC8 and LC9 are enriched for DCL2,3,4 independent sRNA loci, while the remaining PolV-independent class LC6 is significantly depleted for such loci. Conversely, 70% of the loci in the PolV-dependent class LC7 are identified as DCL2,3,4 independent, a highly significant association. There are also possible functional distinctions between the identified locus classes that do not correlate with the major biogenesis pathways; for example, LC1, LC3, LC5, and LC7 all show significant association with gene promoter regions, while LC2, LC6, LC8 and LC9 all show significant depletion in these regions.

The locus classes show a variety of significant overlap with transposable element superfamilies, with the overlaps with RC/Helitron and LTR/Gypsy showing strong correlation between the PolV-dependent and PolV-independent split between locus classes. However, associations with several other types of transposable element superfamiles cross the PolV-dependency division. It is worth noting that overlaps with transposable element superfamily and other annotation features are not used to direct the clustering other than as one factor in the choice of *k*; consequently, the sharp divisions in association between locus classes provide some evidence that these classes have biological meaning.

This analysis of sRNAs and sRNA loci in *Arabidopsis thaliana* is intended to facilitate future analysis of these diverse types of RNA in that model species. Our approach also provides a framework for the analysis of sRNA loci in other plant species.

## Methods

### Datasets used in this study

The libraries are listed in Supplementary Table 1. All libraries were sequenced from tissue from *Arabidopsis thaliana* Col-0 ecotype samples. The segmentation map is based on 98 libraries of wild-type and mutant Arabidopsis samples contained in 54 replicate groups, split by tissue, condition, sequencing author and experiment. To minimize bias, we have chosen only external libraries sequenced by Illumina sequencing machines.

### Published sRNA-Seq datasets

#### Preprocessing, filtering and mapping

All small RNA libraries were processed in the same manner. 3′ adapter trimming was performed using in-house scripts requiring perfect matching; sequences not overlapping with the adaptor on the 3′ end were discarded. The remaining reads were then size selected, keeping only 15–30 nt long reads. These were mapped using PatMaN^33^ against the *Arabidopsis thaliana* genome (TAIR10)^21^, allowing no mismatches and reporting all multimatch locations.

Reads were also aligned to the miRNA hairpin structures in *Arabidopsis thaliana* reported by mirBase^34^, and to tRNA and rRNA sequences reported by TAIR10 in the same manner. Any sequences aligning to miRNA, tRNA or rRNA were removed from the mapping to the genome.

### Small RNA loci

Small RNA loci were identified using the ${\mathtt{segmentSeq}}$ **R** package^20^. Reads mapping to more than one thousand locations on the genome were discarded, as were reads containing contiguous regions of eleven nucleotides in length of which at least ten were the same base. Library scaling factors for each library were calculated as the sum of the lowest 75% of expressed sRNA sequences in that library.

Regions were considered as potential small RNA loci provided they contained no gap of at least one hundred nucleotides in length to which no small RNA aligned in any sample. Initial heuristic segmentation identified loci in each replicate set on the basis of an RPKM of at least one thousand, containing no gap of greater than one hundred nucleotides in which the RPKM fell below this level. Empirical Bayesian estimation of likelihoods that the identified regions constituted an expressed small RNA loci was carried out over three bootstrap cycles. Re-segmentation of the results from the heuristic approach was then carried out based on empirical Bayesian estimates of expression of small RNAs using the default parameter settings of the ${\mathtt{segmentSeq}}$ package. Empirical Bayesian estimation of likelihoods that the newly identified regions constituted an expressed small RNA loci was carried out over three bootstrap cycles as before.

### Computing sRNA locus features

Various studies have examined associations of a range of features associated with sRNA loci with their role in sRNA biogenesis pathways^35,36^. We therefore annotated each sRNA locus with a range of features (see Table 1) in order to categorise the loci. For those features derived directly from the sRNA loci and the sequencing data used to identify them, (e.g. sRNA length, 5′ starting nucleotide frequency and tissue specificity) we based our annotation on data from the wild-type libraries, omitting libraries derived from mutants such as *nrpd1a* or immuno-precipitation (IP) experiments since these libraries do not reflect endogenous sRNA distributions.

While many of the sRNA locus annotation features are categorical in nature, several, such as 21/24 ratio, strand ratio, *et cetera* are continuous. We find that these features are also in general bi- or multi-modal (Supplementary Figures 8–22), reflecting the existence of distinct categories of sRNA loci. For the continuous data, we therefore identified by inspection of the empirical distribution of the data a set of thresholds partitioning the distribution. Where the values of the data are not dependent on the sequencing depth (e.g., locus width), these thresholds were used to partition the data to give the categories described in Table 1. However, several of these features are dependent on the sequencing depth at the locus. For example, the quality of the estimate of the 21/24 ratio at a locus will depend on the total abundance of 21/24 reads. In these cases, a 95% confidence interval on the estimates was derived from the data for a given locus. If this confidence interval lay wholly within an individual partition of the data, the locus was annotated accordingly. If the confidence interval crossed a single threshold, the locus was annotated as belonging to the partition nearest some default value (see Table 1). If the confidence interval crossed more than one threshold, no annotation was recorded for this feature.

### Annotation features

#### sRNA loci size

Loci were split into three bins; those with width greater than 0 nt and less than or equal to 50 nt, those with greater than 50 nt and less than 2000 nt, and those greater than 2000 nt.

#### Expression

Loci were identified as overlapping (at a minimum of a single base) with the set of long-RNA (RNA-Seq) expression loci defined by Luo *et al*.^22^. This set of expression loci was chosen to correlate with the histone modification data described below.

#### Phasing

The presence of phasing was assessed at each locus using the phaseR tool (https://github.com/bacms2/phaser) for each wild-type experimental group in the data. For any given locus, phasing was identified as ‘moderate’ if statistically significant (0.05 level) in at least one experimental group, and ‘high’ if statistically significant in more than 5 experimental groups.

### Small RNA 21/24 ratio

The ratio of 21-22 nt small RNAs to 23-24 nt small RNAs at a given locus, referred to here as the 21/24 ratio, is well established as indicative of the function of the sRNAs at that locus. Post-transcriptional gene silencing (PTGS) sRNAs, including miRNAs, ta-sRNAs/phasRNAs, vi-RNAs and hpRNAs are typically 21/22 nucleotides long whereas sRNAs involved in RNA directed DNA methylation (RdDM) are generally 23/24 nucleotides and referred to as hc-sRNAs^8,11^.

The precision with which this ratio can be calculated is highly dependent on the depth of coverage at a given locus. We therefore estimated confidence intervals on the ratio assuming a binomial distribution, and allocated the locus to one of three bins describing a low, moderate and high proportion of 21 s as described above. Thresholds for the bins were chosen by examining density plots of this ratio on a logarithmic scale (Supplementary Figure 8).

### Tissue

The ‘Tissue’ classification refers to the ubiquity of sRNA expression across diverse tissue types. If the likelihood that a locus was expressed was greater than 50% in at least ten wild-type replicate groups, the locus was classed as ‘common’, if in more than one but less than ten wild-type replicate groups, the locus was classed as ‘intermediate’, and if in one or fewer wild-type replicate groups, the locus was classed as ‘specific’.

### Repetitiveness

Repetitiveness is a measure of the extent to which small RNAs that align to a given locus may also align to other genomic locations. We assess this at each locus as

1 $$R=1-\sum _{i}\frac{{x}_{i}}{{m}_{i}}/\sum _{i}{x}_{i}$$

where *x*_*i*_ is the number of times the *i* th small RNA within the locus is sequenced and *m*_*i*_ is the number of genomic locations to which that small RNA aligns. Thus, if all small RNAs at a given locus align to that locus and to no other location on the genome, *R* will be zero. If the number of alternate locations to which the small RNAs align is high, *R* will be close to 1. Since the precision with which the repetitiveness ratio can be calculated is dependent on the depth of coverage at that locus, we estimated confidence intervals on this assuming a binomial distribution, and allocated the locus to one of three bins describing a low, moderate and high repetitiveness as described above. Thresholds for the bins were chosen by examining density plots of this ratio (Supplementary Figure 9).

### Strand ratio

Strand ratio is a measure of the bias at a given sRNA locus for the sequenced small RNAs to derive predominantly from one strand of DNA. We evaluated this by considering the ratio of sRNAs aligning to the positive strand to those aligning on the negative strand. Since the measurement of this bias will depend on the coverage, we estimated confidence intervals on this assuming a binomial distribution, and allocated the locus to one of five symmetrically distributed bins describing a high, moderate, low, moderate and high strand bias as described above. Thresholds for the bins were chosen by examining density plots of this ratio (Supplementary Figure 10).

### DCL2/3/4 dependency

The dependency of a sRNA locus’ expression on the DCL2/3/4 proteins was evaluated using the mutant DCL2/3/4 line SL300 detailed in Supplementary Table 1. Using the estimated likelihoods of locus expression for this line, a set of 6868 loci were selected as expressed in the DCL2/3/4 mutant with a false discovery rate of 0.05. These were characterized as DCL2/3/4 independent; all other loci were characterized as DCL2/3/4 dependent.

### RDR2/PolIV/PolV dependency

The dependency of a sRNA locus’ expression on RDR2, PolIV and PolV was evaluated based on comparisons between the wild-type inflorescence samples from Lee^37^ and the mutant samples from the same study. Differential expression between the wild-type samples and the individual mutant samples was evaluated using baySeq^38^. Those sRNA loci that show a loss of expression in the mutant line with a false discovery rate of less than 0.05 are identified as dependent on that mutant, while the remainder of the loci are identified as not dependent on that mutant.

### Cytosine methylation (CpG/CHG/CHH)

The level of cytosine methylation in the three contexts CpG, CHG and CHH was evaluated based on the processed wild type data from Stroud^39^. This data consists of proportions of methylation evaluated at each cytosine. For each sRNA locus and context of methylation we identified the set of cytosines from the appropriate context that overlap the given sRNA locus. We bootstrapped 95% confidence intervals on the mean methylation at the locus from the set of values identified as the proportion of methylation at the given locus. We then assigned the loci to a set of bins chosen by observation of the distribution of the mean methylation at each locus (see Supplementary Figures 11–13) as described above.

### Histone accumulation (H3)

ChIP-Seq data were obtained from Luo *et al*.^22^ and remapped to the Arabidopsis genome using bowtie using the following parameters:

$$-{\rm{C}}-\,{\rm{a}}-\,{\rm{m}}\,1-\,{\rm{v}}\,1-\,{\rm{q}}\,\${\rm{base}}.{\rm{fastq}}-\,{\rm{S}}\,\${\rm{base}}.{\rm{sam}}.$$

We then identified the number of sequenced reads in the histone chIP data that overlap each sRNA locus. To estimate rates of H3 accumulation at each locus, we estimate the rate per base and 95% confidence intervals on this rate by assuming a Poisson distribution on the number of reads, with rate proportional to the width of the locus. The loci were then assigned to a set of bins chosen by observation of the distribution of the logarithm of the rate per base of histone accumulation over the sRNA loci (see Supplementary Figure 14).

### Histone marks

The presence of individual histone marks at each locus was evaluated for each mark by considering the ChIP-Seq data acquired from Luo *et al*.^22^ and mapped to the Arabidopsis genome as above. The number of sequenced reads for the given mark overlapping a given locus was compared to the number of sequenced reads in the H3 dataset overlapping that locus. It was assumed that these data are distributed binomially, and 95% confidence intervals on the proportion of overlapping reads derived from the given mark were estimated and used to assign the loci to a set of bins chosen by observation of the distribution of the estimated proportions at each locus (see Supplementary Figures 15–22).

### 5′ base preference

Of the small RNA sequences deriving from the wild-type samples described in Supplementary Table 1 with width between 21–24 bases which align to the genome, the 5′ base is A for 51% of the sequences, C for 9% of the sequences, G for 17% of the sequences and T for 22% of the sequences.

At each locus, we compared the proportion of wild-type sRNAs whose 5′ base is A, C, G and T to that of all wild-type sRNAs aligning to the genome, assuming a binomial distribution. We computed p-values on the observed data and adjusted for multiple testing, following Benjamini^40^. If the adjusted p-value for a given locus and nucleotide showed significant enrichment of that nucleotide relative to the genome-aligning population of sRNAs, we recorded that locus as showing a preference for that nucleotide at the 5′ base of the sRNAs. Consequently, it is possible for a given locus to show a preference for up to three different nucleotides at the 5′ base; for example, an individual sRNA locus may be significantly enriched in sRNAs with A, C, and T at the 5′ base.

### Feature overlap

We evaluated feature overlap by considering any overlap (i.e., one or more bases overlap) between the coordinates of the sRNA loci and reported features.

We considered overlap with gene, ncRNA, pseudogene, rRNA, tRNA features as reported in the TAIR10 annotation^41^. We also considered overlap with TE superfamily members DNA, DNA/Harbinger, DNA/HAT, DNA/Mariner, DNA/MuDR, DNA/Pogo, DNA/Tc1, LINE, LINE/L1, LTR/Copia, LTR/Gypsy, RathE, RC/Helitron and SINE elements, also as reported in the TAIR10 annotation^41^.

We also considered overlap with natural antisense transcripts (NATs)^22^, long intergenic non-coding RNAs (lincRNAs)^23^ and epigenetically activated siRNAs (easiRNAs)^12^.

### Inverted repeats

Inverted repeats (IR) can be processed to sRNAs, as shown for IR71 and IR2039^32^. To this end, we computed genome-wide putative IRs employing inverted repeat finder (IRF) version 3.05^42^ using the following parameters:

$${\rm{wine}}\,{\rm{irf}}305\,.{\rm{dos}}\,.{\rm{exe}}\,{\rm{arabidopsis}}\,.{\rm{fa}}2\,3\,5\,80\,10\,40\,500000\,10000-{\rm{d}}-\,{\rm{h}}-\,{\rm{t}}4\,74-\,{\rm{t}}5\,493-\,{\rm{t}}7\,10000$$

The coordinates acquired from these predictions were used to annotate each sRNA locus as overlapping an IR locus, or not.

### Dimension and cluster selection

MCA is a dimension reduction technique in which categorical data are transformed along orthogonal axes with an associated eigenvalue defining the percentage of variation explained by each dimension. In order to maximise the performance of subsequent unsupervised learning on the transformed data, it is necessary to define a cutoff at which dimensions explaining a low percentage of variation are discarded. We do this by considering the summary data shown in Supplementary Figure 7A,B. Supplementary Figure 7A shows the (ranked) percentage of variance explained by each dimension; there is a clear ‘elbow’ in the percentage of variation explained between the 4th and 6th dimensions. Supplementary Figure 7B shows the stability of the clusterings achieved for all combinations of dimension selection from 1–8 and all numbers of clusters from 2–10. Stability is calculated by randomly sampling (with replacement) from the methylation loci a set identical in size to the original. After re-analysis of the sampled data, we observed for each cluster the proportion of the sampled data which preserved its clustering. By repeating this sampling multiple times we were able to create boxplots of the distribution of cluster stability. We thus observe that if four or five dimensions of the data are used, the clustering is generally unstable for more than four clusters. If six or more dimensions are used, however, a nine cluster solution is stable, allowing greater separation of the feature space.

Given a reduction of the data to six dimensions, we established the correct number of clusters by calculating Tibshirani’s gap statistic^25^, in which we sought to maximise the difference between the observed and expected sum-of-squares within each cluster relative to the cluster means. (Supplementary Figure 7C). This indicated either 9 or 11 clusters might be appropriate; we chose nine as the more conservative estimate. We also computed the normalised mutual information (NMI) comparing the clustering to transposable element superfamily overlap and feature overlap (Supplementary Figure 7D), which indicated NMI is maximised for nine clusters, though remains high for ten and eleven cluster solutions.

### Accession codes

Sequencing data are made available through the European Nucleotide Archive (ENA)(http://www.ebi.ac.uk/ena) under accession number PRJEB18944. Identified loci are also available on the ENA under accession number PRJEB22276.

## Electronic supplementary material

Supplementary Material
