# Supplementary material for: Towards annotating the plant epigenome: the Arabidopsis thaliana small RNA locus map
Source: Sci Rep. 2018 Apr 20;8:6338. doi: 10.1038/s41598-018-24515-8 (PMC5910406; doi:10.1038/s41598-018-24515-8)
Supplement: Supplementary file 1 — Supplementary Material [file 41598_2018_24515_MOESM1_ESM.pdf]

Towards annotating the plant epigenome: the Arabidopsis thaliana small RNA locus map
Supplementary Material

Hardcastle, Thomas J.[1] Mueller, Sebastian Y.[1]
Baulcombe, David C.[1,2]

March 6, 2018

[1]Department of Plant Sciences, University of Cambridge, Downing Street, Cambridge CB2 3EA, United Kingdom
[2]To whom correspondence should be addressed.

Supporting Information (SI)

Tissue	Plant/Experiment Type	Library ID	Notes	Library size	Library scaling factor
Arial	AGO4 IP	SL26		1199099	378874
Arial	WT	SL2013		10425726	1014040
Arial	WT	SL2014		9471287	994804
Floral	AGO1 IP	SL34		2055185	128529
Floral	AGO1 IP	SL48		2016920	69785
Floral	ago4-3	SL25_1 [Havecker et al., 2010]		1365121	381754
Floral	ago4-3	SL25_2 [Havecker et al., 2010]		137269	62558
Floral	ago4-3	SL25_3 [Havecker et al., 2010]		675977	214460
Floral	AGO4 IP	SL32 [Havecker et al., 2010]		1247107	349998
Floral	AGO4p:AGO4 IP	SL234_B [Havecker et al., 2010]	tech rep SL234_B	475851	216458
Floral	AGO4p:AGO4 IP	SL234_B_2 [Havecker et al., 2010]	tech rep SL234_B	3029251	1011738
Floral	AGO4p:AGO6 IP	SL234_C [Havecker et al., 2010]	tech rep SL234_C	361231	164966

Floral	AGO4p:AGO6 IP	SL234_C_2 [Havecker et al., 2010]	tech rep SL234_C	2161880	731039
Floral	AGO4p:AGO9 IP	SL234_F [Havecker et al., 2010]		883258	313942
Floral	AGO5 IP	SL103_2 [Havecker et al., 2012]	tech rep 103	317109	61955
Floral	AGO6 IP	SL10		1109393	398932
Floral	JAM51	SL41		533800	185674
Floral	JAP3	SL40		1249490	309729
Floral	nrpd1a	SL168 [Mosher et al., 2008]		226682	32263
Floral	+/nrpd1a	SL170 [Mosher et al., 2008]		322948	87570
Floral	nrpd1a/+	SL169 [Mosher et al., 2008]		365311	100900
Floral	nuclear	SL383_D [Mosher et al., 2008]		1796412	407099
Floral	nuclear	SL383_E [Mosher et al., 2008]		345882	99362
Floral	WT	SL167 [Mosher et al., 2008]		419591	122767
Floral	WT	SL362		17587390	2076076
Floral	WT	SL363		17169771	2415846
Floral	WT	SL382_B		821403	187866

Floral	WT	SL382_G		3100603	342370
Floral	AGO1 IP	SL77		2116966	134637
Floral	WT	SL9		1031870	347321
Floral A	WT	SL23_1	tech rep SL23	198190	100156
Floral A	WT	SL23_2	tech rep SL23	452839	199780
Floral A	WT	SL5		421535	159321
Floral B	WT	SL334		393175	139032
Floral B	WT	SL335		336821	107707
Floral B	WT	SL99		907317	251453
Floral	JAM39	SL100		674503	207397
Floral/Silique	AGO1 IP	SL35		1783258	74888
Floral/Silique	AGO1 IP	SL49		1221299	57445
Floral/Silique	AGO9 IP	SL11		1464268	216026
Floral/Silique	AGO9 IP	SL12		2104362	263745
Leaf	AGO1 IP	SL44_1		1840510	24598

Leaf	AGO1 IP	SL50		594305	23151
Leaf	dcl4-2; AGO1 IP	SL47		1212192	55981
Leaf	nuclear	SL381_C [Mosher et al., 2008]		2740535	428989
Leaf	nuclear	SL383_F [Mosher et al., 2008]		438215	109215
Leaf	rdr6-15; AGO1 IP	SL46		1477080	18943
Leaf	rdr6-15; AGO1 IP	SL52		275990	12644
Leaf	tex1-1; AGO1 IP	SL45		1745726	19506
Leaf	tex1-1; AGO1 IP	SL51		924476	33223
Leaf	WT	SL161		829213	157691
Leaf	WT	SL381_A [Mosher et al., 2008]		2858168	231195
Leaf	WT	SL382_H [Mosher et al., 2008]		201469	42305
Root	Col/Col	SL258 [Molnar et al., 2010]		6959763	835320
Root	Col/Col	SL259 [Molnar et al., 2010]		6103188	566694
Root	dclTriple/dclTriple	SL236 [Molnar et al., 2010]		3553900	130715
Root	dclTriple/dclTriple	SL260 [Molnar et al., 2010]		8777502	270427

Seedling	WT	SL115		2033096	451640
Seedling	JAM39	SL97		2338603	246677
Seedling	JAM94	SL98		2535338	444023
Shoot	Col/Col	SL302		9697701	1384093
Shoot	Col/Col	SL303		9984519	1593634
Shoot	dclTriple/dclTriple	SL300		8411664	896231
Shoot	dclTriple/dclTriple	SL301		7837710	878581
Silique	WT	SL90_1 [Mosher et al., 2009]		139388	46391
Floral/Silique	WT	SL4_1		2572837	510957
Floral/Silique	WT	SL4_2		3255817	770543
Unknown	AGO2 IP	SL28_1	tech rep SL28	236226	91063
Unknown	AGO2 IP	SL28_2	tech rep SL28	59510	29377
Unknown	AGO2 IP	SL28_3	tech rep SL28	39535	18851
Unknown	AGO2 IP	SL28_4	tech rep SL28	1386968	430946
Unknown	AGO2 IP	SL29_1	tech rep SL29	166638	65603

Unknown	AGO2 IP	SL29_2	tech rep SL29	1172375	283978
Unknown/leaf?	AGO1 IP + mock	SL206		3970841	131601
Unknown/leaf?	AGO1 IP + TCV	SL205		3292945	211703
Unknown/leaf?	AGO2 IP + mock	SL208		3573129	658133
Unknown/leaf?	AGO2 IP + TCV	SL207		1166761	204939
Floral A	WT	SL30_1 [Hardcastle et al., 2012]	tech rep SL30	348278	125966
Floral A	WT	SL30_2 [Hardcastle et al., 2012]	tech rep SL30	237542	102825
Floral A	WT	SL30_4 [Hardcastle et al., 2012]	tech rep SL30	2194462	700934
Floral A	WT	SL31 [Hardcastle et al., 2012]		339166	113765
Seedling	WT	SL68		2187150	387020
Seedling	WT	SL69		1960675	315092
Seedling	WT	SL96		4187538	378538
Arial	WT	GSM506656 [Garcia-Ruiz et al., 2010]		683946	257180
Arial	WT	GSM506657 [Garcia-Ruiz et al., 2010]		683946	231327
Arial	WT	GSM506658 [Garcia-Ruiz et al., 2010]		632624	227082

Arial	dpi10	GSM506662 [Garcia-Ruiz et al., 2010]		535229	267888
Arial	dpi10	GSM506663 [Garcia-Ruiz et al., 2010]		675808	299414
Arial	dpi10	GSM506664 [Garcia-Ruiz et al., 2010]		821979	757362
Inflorescence	WT	GSM893112 [Lee et al., 2012]		1974218	600576
Inflorescence	WT	GSM893113 [Lee et al., 2012]		2117298	2104335
Inflorescence	WT	GSM893114 [Lee et al., 2012]		7605356	1740866
Inflorescence	WT	GSM342999 [Montgomery et al., 2008]		6228375	809366
Inflorescence	WT	GSM343000 [Montgomery et al., 2008]		2636014	591566
Inflorescence	WT	GSM343001 [Montgomery et al., 2008]		2453210	539950
Shoot	Salt stress	GSM832032 [Zhang et al., 2012]		2481592	3283668
Shoot	Salt stress	GSM832033 [Zhang et al., 2012]		16238137	734404

Table 1: **siRNA libraries**. The siRNA sequencing libraries used to define sRNA loci and characterise their behaviour. Where these libraries have previously appeared in published work the primary citation is noted. Library IDs with the prefix SL are taken from internal tracking, whereas those with prefix GSM are from the Gene Expression Omnibus **?**. All internal libraries have been released through the European Nucleotide Archive (ENA) (http://www.ebi.ac.uk/ena) under accession number PRJEB18944.

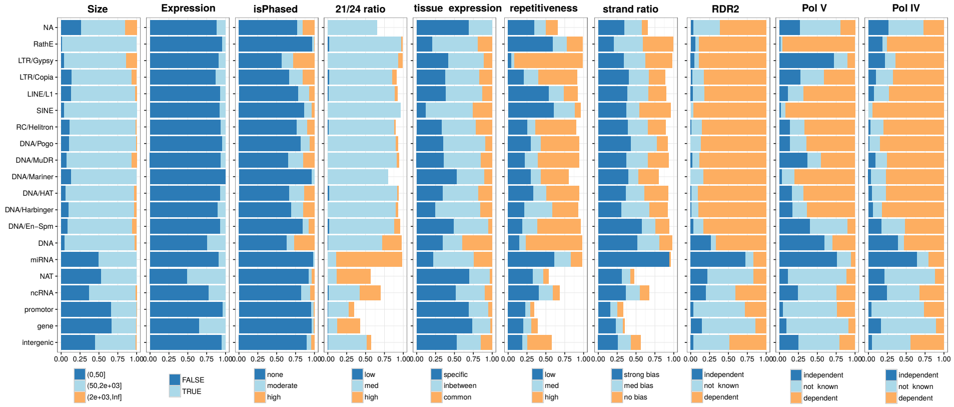

Figure 1: **Different elements are known to be involved in different sRNA biogenesis pathways.** Vertical panels represent individual features. Each locus is allocated to categorical classes as described in Methods (white areas indicate that the feature state could not be determined). To demonstrate the potential for these features to differentiate sRNA locus classes, feature classes statistics were broken down by their overlap with genetic elements (y-axis). The relative fraction of loci for each feature state (described in Methods) is shown on the bar plots.

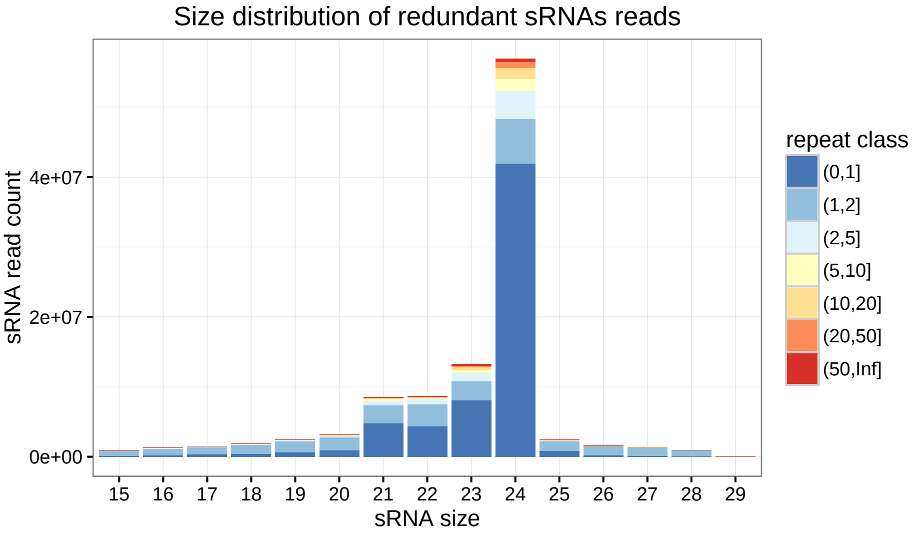

Figure 2: **Length distribution of small RNAs**. The y-axis corresponds to the read counts of redundant processed but unfiltered sRNAs across all wild type libraries. 24nt long sRNAs are most abundant (54,2% of of total reads) followed by 23nt (12,6%), 22nt (8,3%) and 21nt (8,1%). The colour code corresponds to sRNA repetitiveness i.e. the number of times a particular sRNAs species maps to the genome. Most reads (60%) map uniquely to the genome (dark blue), but the proportion varies within size classes with 74% unique reads for 24nt long sRNAs, whereas only about 55% of the 21nt long sRNAs are unique.

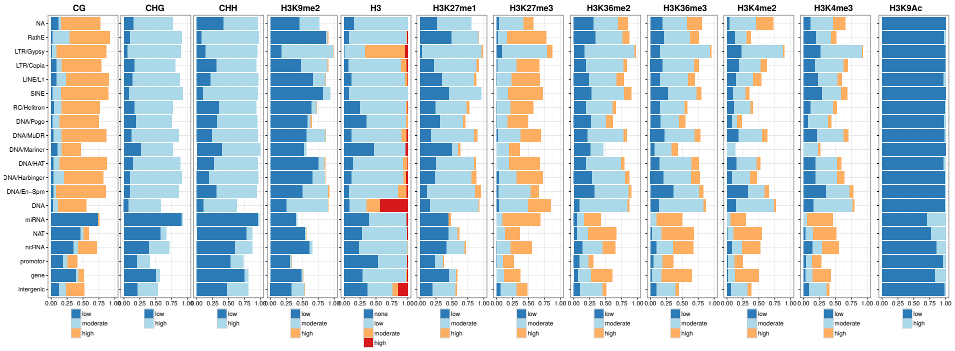

Figure 3: Vertical panels represent individual epigenetic features. Each locus is allocated to categorical classes as described in method section. To demonstrate discriminate power, feature classes statistics were broken down by genetic elements (y-axis).

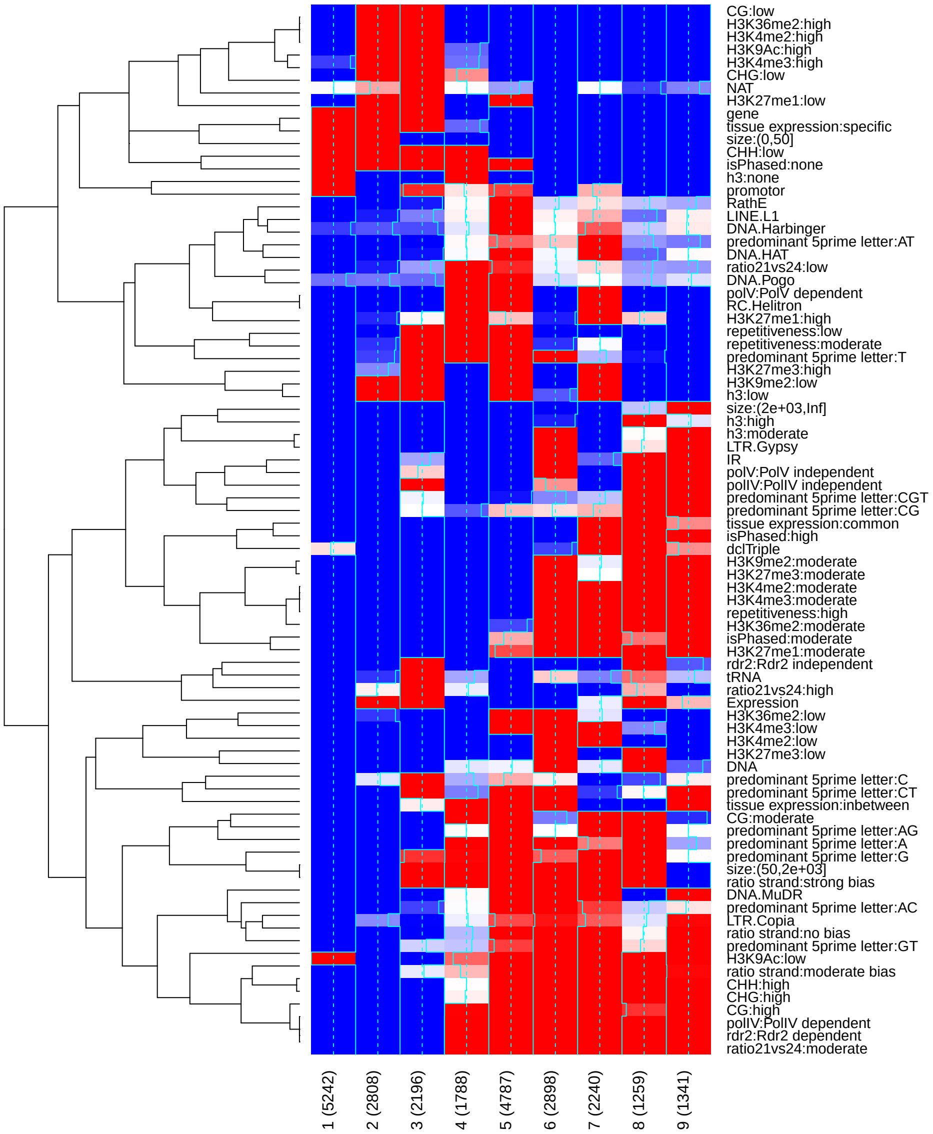

Figure 4: Enrichment of association for all significant features in each locus class. Red indicates positive and blue negative association with the intensity of the colour measuring the level of significance.

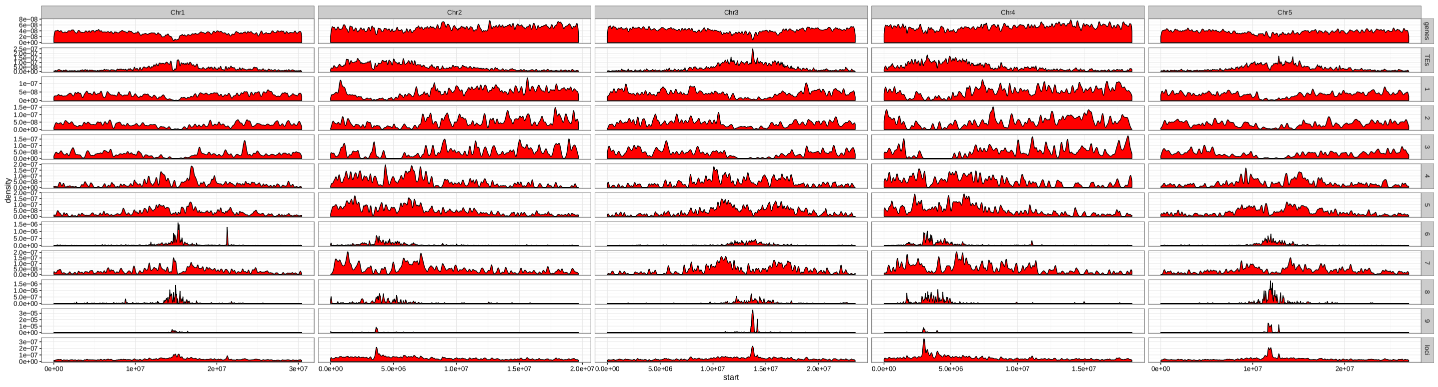

Figure 5: **Genome wide sRNA class profiles.** sRNA cluster profiles for all 5 chromosomes analogous to Figure 5.

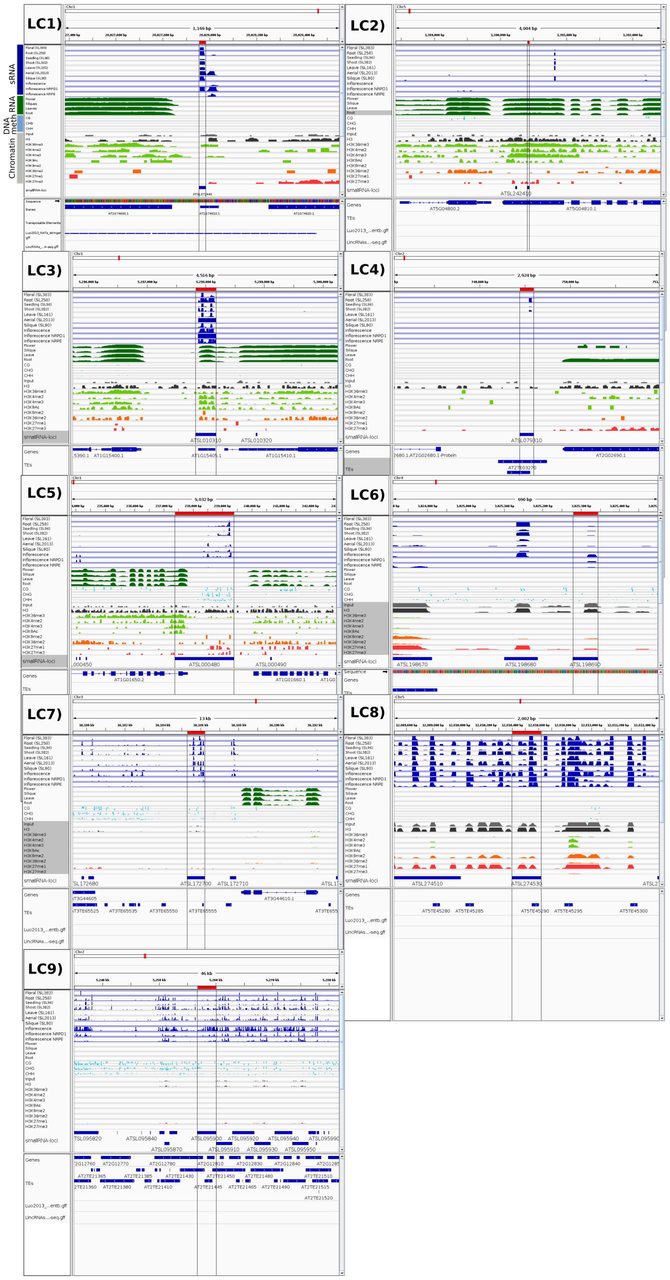

Figure 6: **Genome browser view for all LC paragons analogous to Figure 8.** Top tracks (blue) depict sRNA coverage for a subset of libraries used in this study. The green tracks below show wild-type RNA-Seq libraries for various tissues obtained from Liu et al. [2012] which is followed by wild-type DNA-methylation tracks for CG, CHH and CHG contexts obtained from Stroud et al. [2014]. Histone marks are shown further below indicated with a grey box and subdivided into light green, orange and red tracks reflecting their repressive associations with red being repressive and green indicating active marks. Data were obtained from Liu et al. [2012]. sRNA loci are shown on track below with individual loci shown as blue bars.

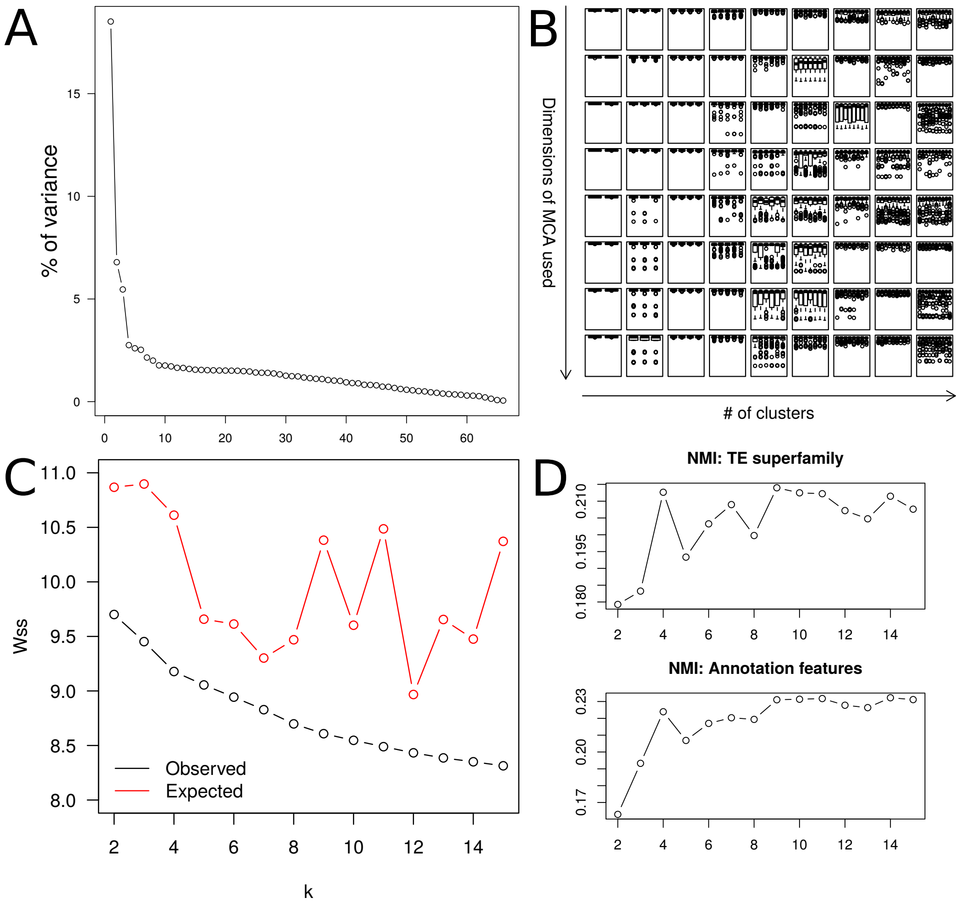

Figure 7: Criteria used to select dimension and number of clusters used to partition the MCA transformed data. (A) shows the ranked percentage of variance explained by each dimension of the MCA transformed data. (B) shows the stability of the clusterings (under bootstrapped sampling) achieved for all combinations of dimension selection from 1-8 and all numbers of clusters from 2-10. (C) shows the observed and expected sum-of-squares within each cluster relative to the cluster means, following Tibshirani et al. [2001]. (D) shows normalised mutual information (NMI) comparing the clustering to transposable element superfamily overlap and feature overlap.

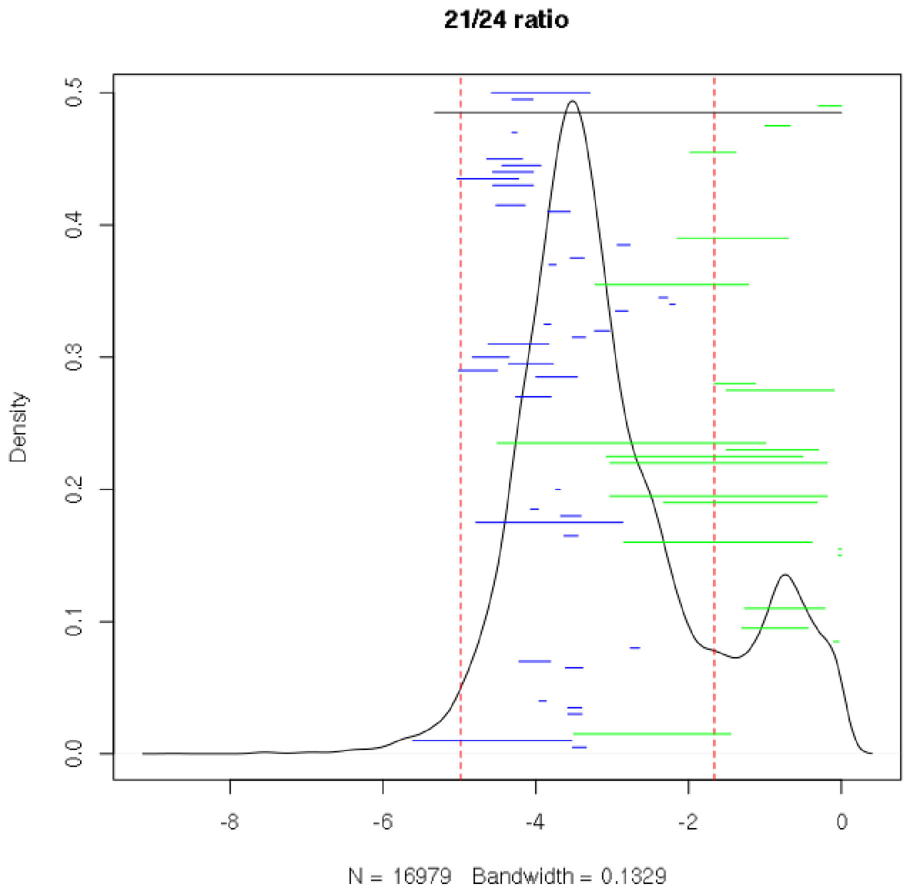

Figure 8: Density plot of the $\log_2$ of the 21/24 ratio at each locus. Thresholds for binning the loci are indicated by the red dashed lines. Also shown are horizontal segments describing the confidence intervals for a small number of loci, colour-coded by the bin to which they are allocated; green indicates a high 21/24 ratio, blue a moderate 21/24 ratio, and red a low 21/24 ratio, while black indicates that the locus cannot be classified.

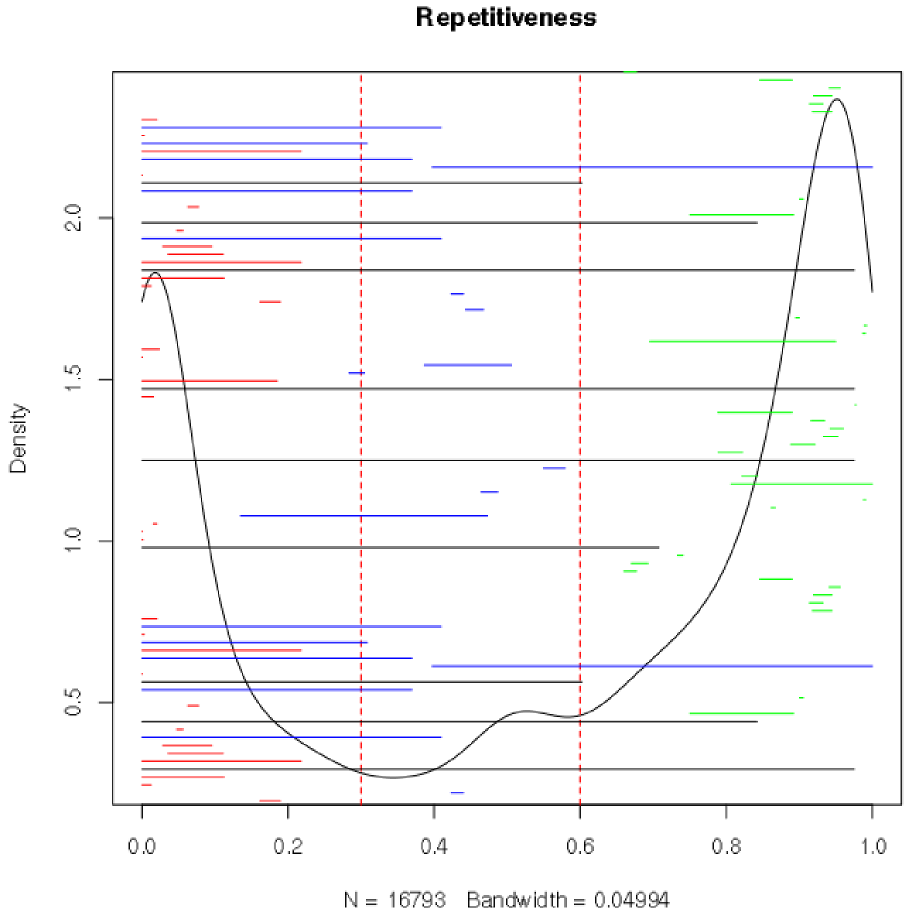

Figure 9: Density plot of the repetitiveness ratio at each locus. Thresholds for binning the loci are indicated by the red dashed lines. Also shown are horizontal segments describing the confidence intervals for a small number of loci, colour-coded by the bin to which they are allocated; green indicates a high repetitiveness ratio, blue a moderate repetitiveness ratio, and red a low repetitiveness ratio, while black indicates that the locus cannot be classified.

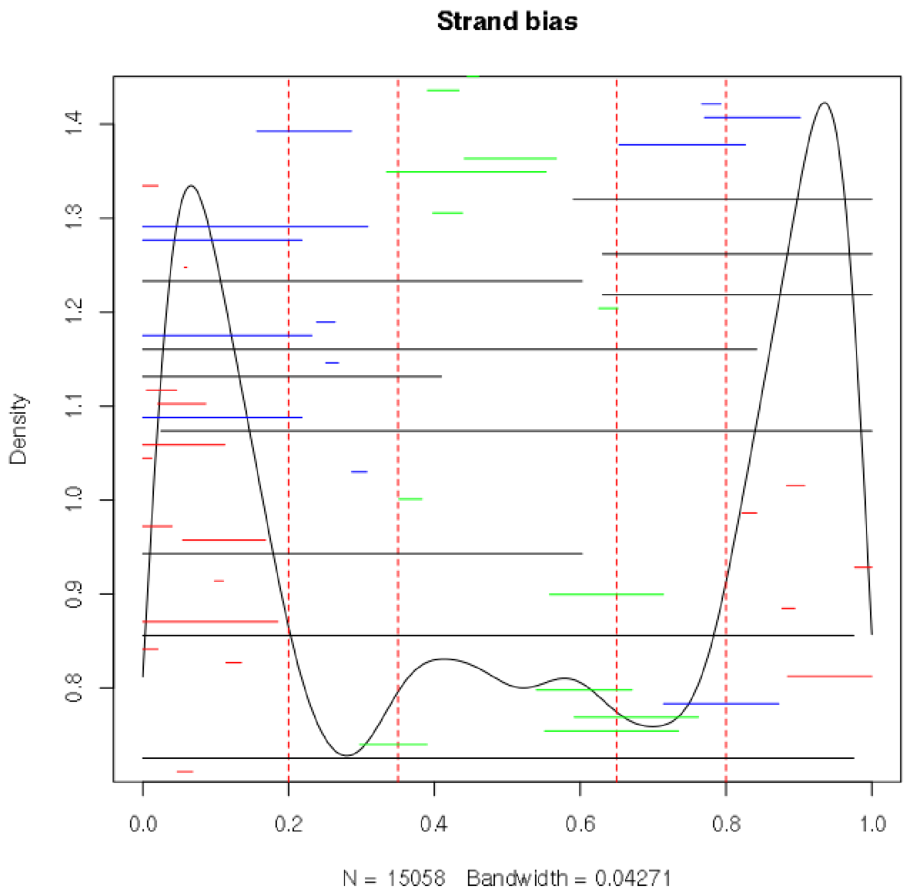

Figure 10: Density plot of the strand bias ratio at each locus. Thresholds for binning the loci are indicated by the red dashed lines. Also shown are horizontal segments describing the confidence intervals for a small number of loci, colour-coded by the bin to which they are allocated; green indicates no strand bias, blue a moderate strand bias ratio, and red a high strand bias, while black indicates that the locus cannot be classified.

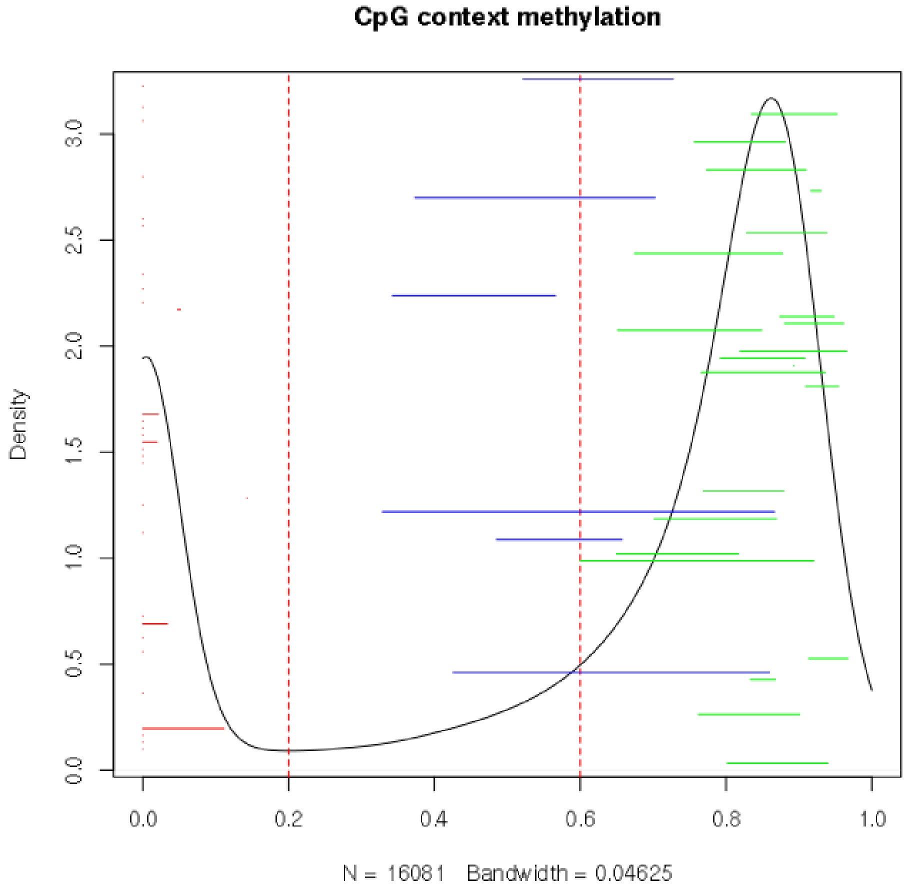

Figure 11: Density plot of the average CpG methylation at each locus. Thresholds for binning the loci are indicated by the red dashed lines. Also shown are horizontal segments describing the confidence intervals on the mean CpG methylation for a small number of loci, colour-coded by the bin to which they are allocated; red indicates low methylation, blue moderate methylation, and green high methylation.

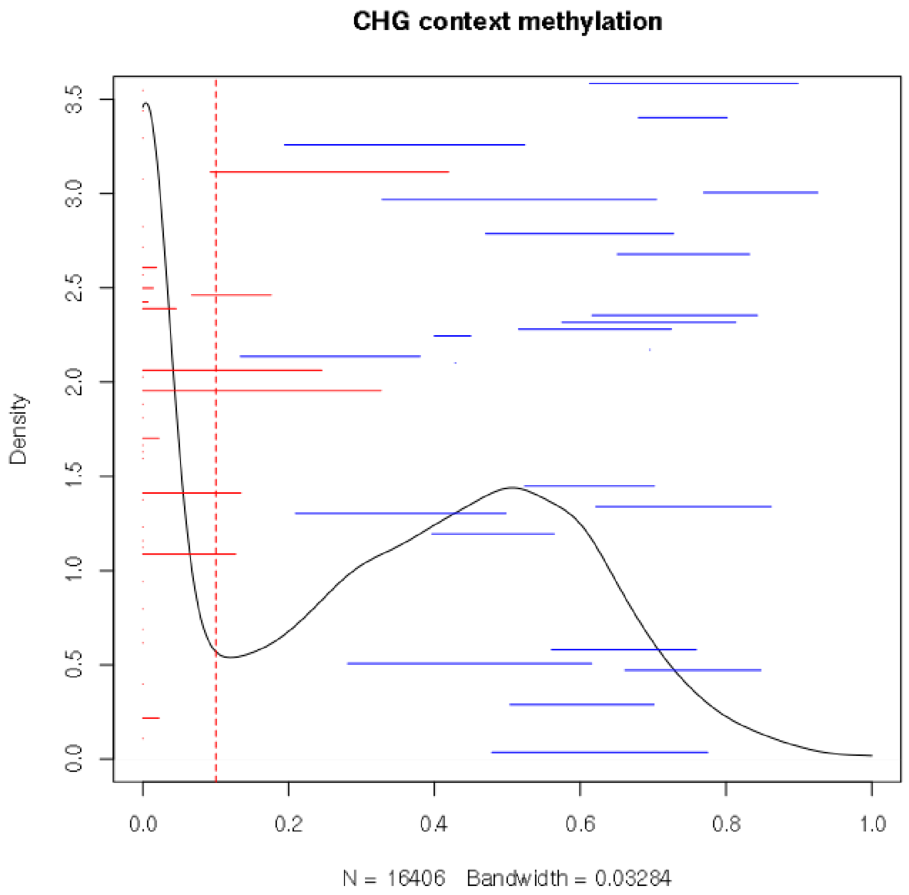

Figure 12: Density plot of the average CHG methylation at each locus. Thresholds for binning the loci are indicated by the red dashed lines. Also shown are horizontal segments describing the confidence intervals on the mean CHH methylation for a small number of loci, colour-coded by the bin to which they are allocated; red indicates low methylation and blue high methylation.

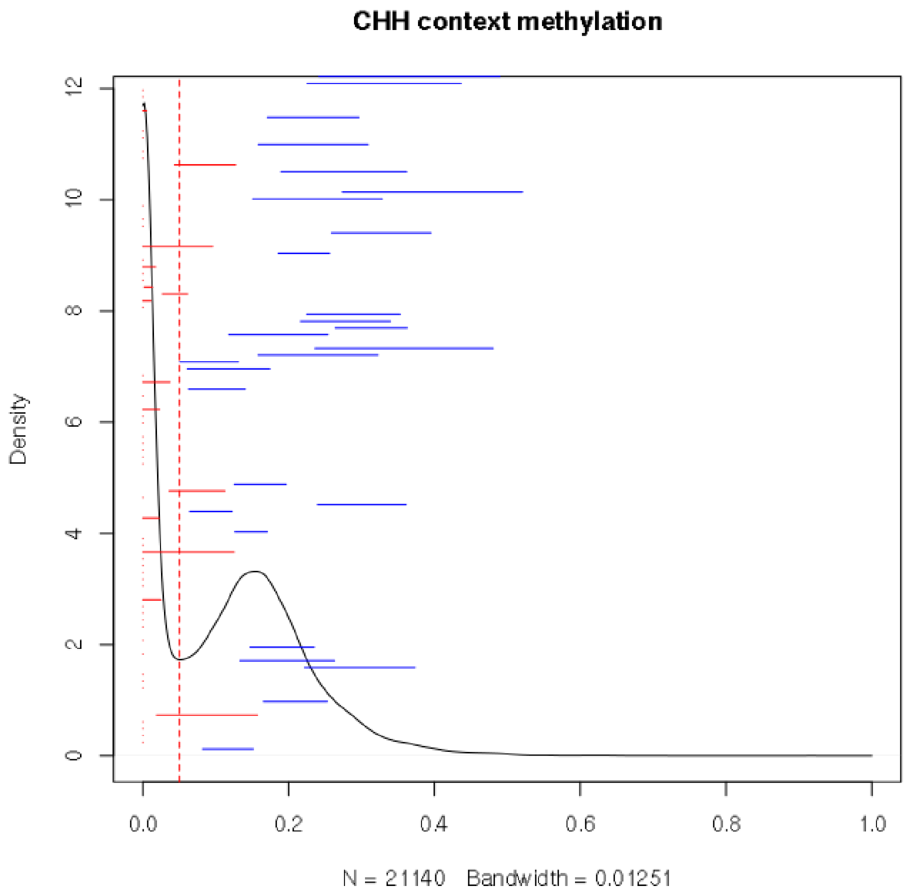

Figure 13: Density plot of the average CHH methylation at each locus. Thresholds for binning the loci are indicated by the red dashed lines. Also shown are horizontal segments describing the confidence intervals on the mean CHH methylation for a small number of loci, colour-coded by the bin to which they are allocated; red indicates low methylation and blue high methylation.

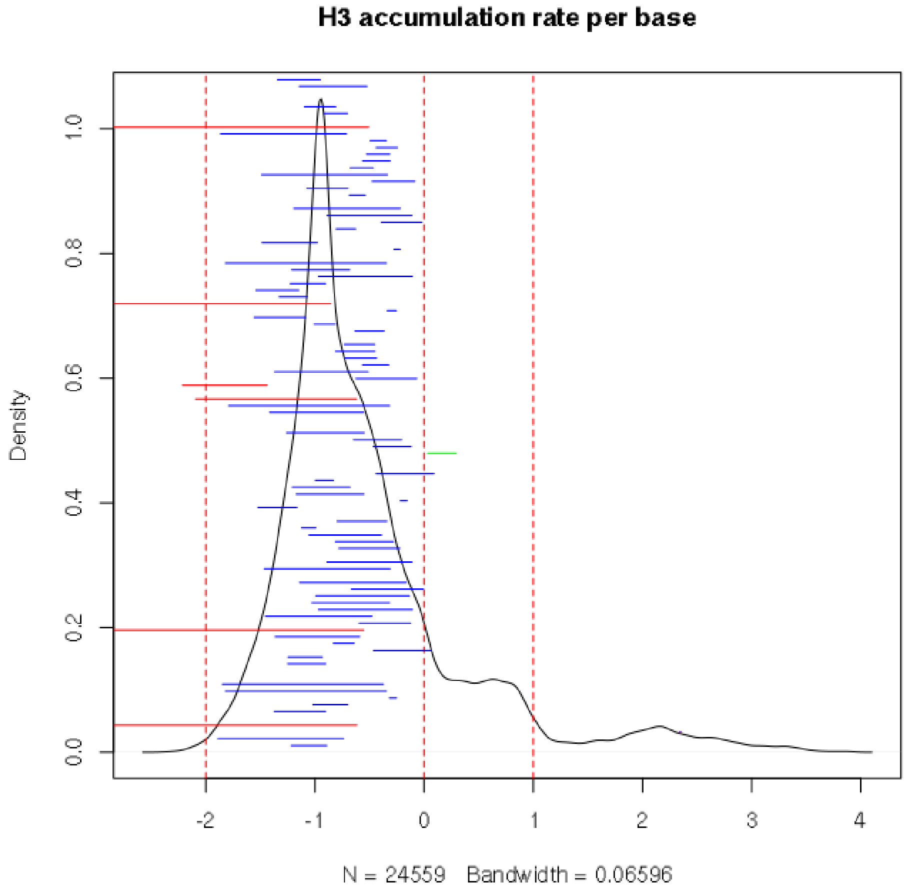

Figure 14: Density plot of the logarithm (base 10) of the average histone accumulation rate at each sRNA locus. Thresholds for binning the loci are indicated by the red dashed lines. Also shown are horizontal segments describing the confidence intervals on the mean histone accumulation for a small number of loci, colour-coded by the bin to which they are allocated; red indicates low H3 presence, blue moderate H3 presence, and green high H3 presence.

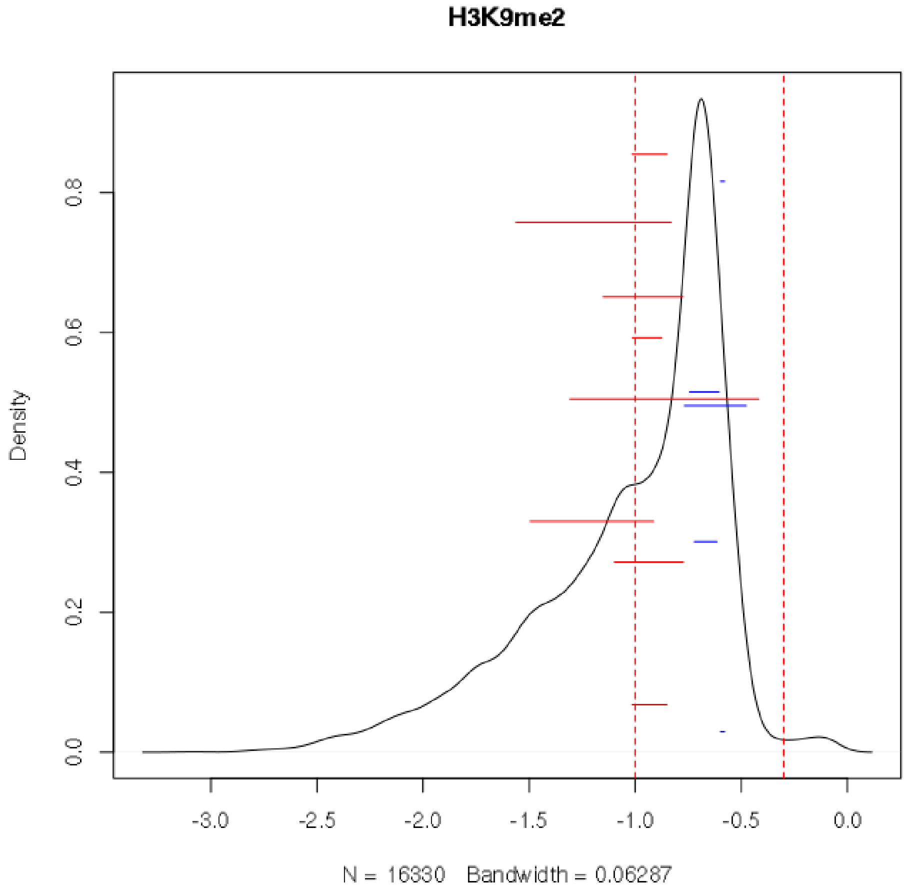

Figure 15: Density plot of the logarithm (base 10) of the proportion of H3K9me2 reads to the total number of H3K9me2 and H3 sequenced reads at each locus. Thresholds for binning the loci are indicated by the red dashed lines. Also shown are horizontal segments describing the confidence intervals on the proportion of H3K9me2 reads for a small number of loci, colour-coded by the bin to which they are allocated; red indicates low H3K9me2 presence, blue moderate H3K9me2 presence, and green high H3Kme2 presence.

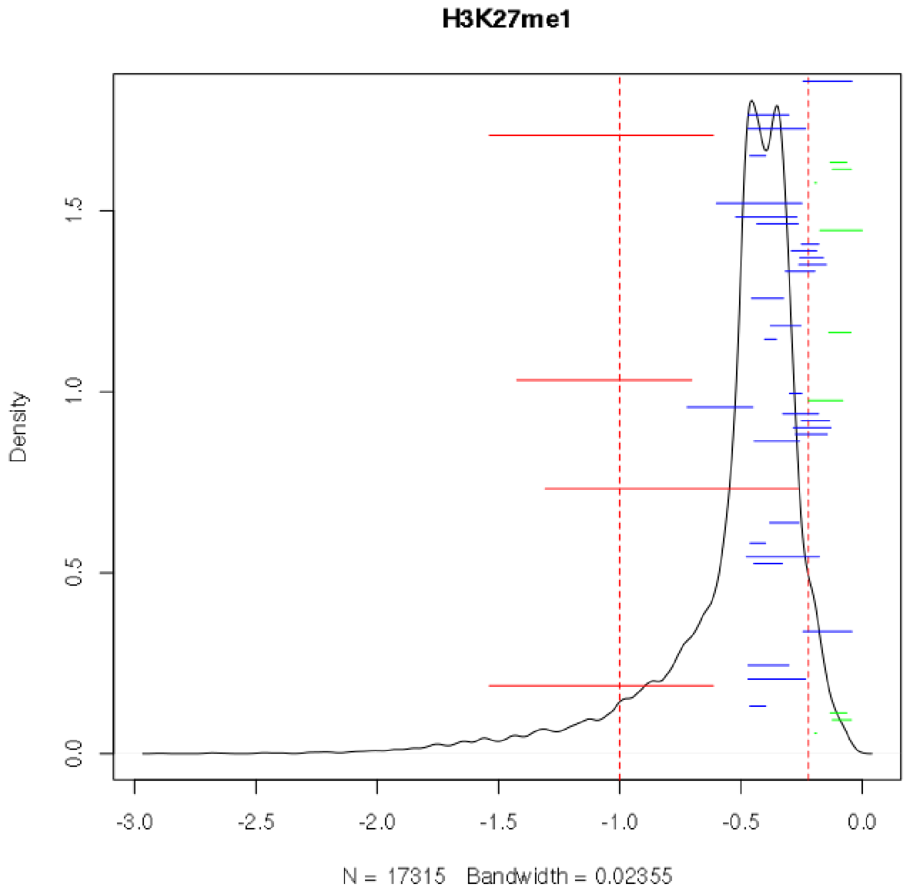

Figure 16: Density plot of the logarithm (base 10) of the proportion of H3K27me1 reads to the total number of H3K27me1 and H3 sequenced reads at each locus. Thresholds for binning the loci are indicated by the red dashed lines. Also shown are horizontal segments describing the confidence intervals on the proportion of H3K27me1 reads for a small number of loci, colour-coded by the bin to which they are allocated; red indicates low H3K27me1 presence, blue moderate H3K27me1 presence, and green high H3K27me1 presence.

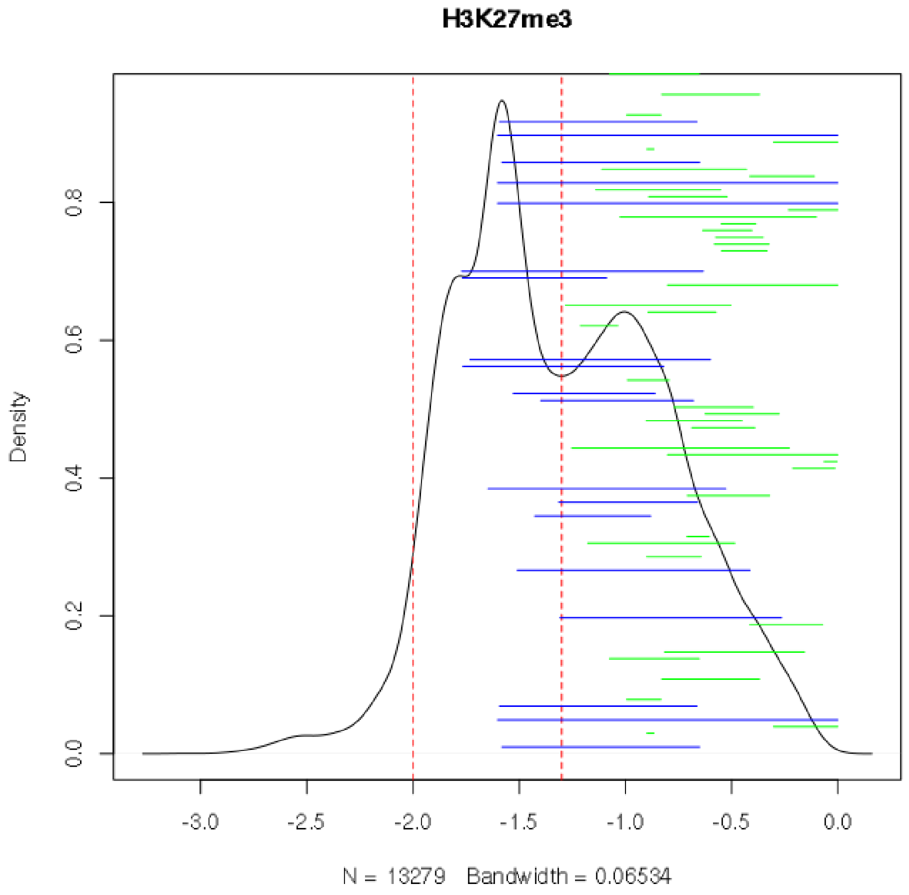

Figure 17: Density plot of the logarithm (base 10) of the proportion of H3K27me3 reads to the total number of H3K27me3 and H3 sequenced reads at each locus. Thresholds for binning the loci are indicated by the red dashed lines. Also shown are horizontal segments describing the confidence intervals on the proportion of H3K27me3 reads for a small number of loci, colour-coded by the bin to which they are allocated; red indicates low H3K27me3 presence, blue moderate H3K27me3 presence, and green high H3K27me3 presence.

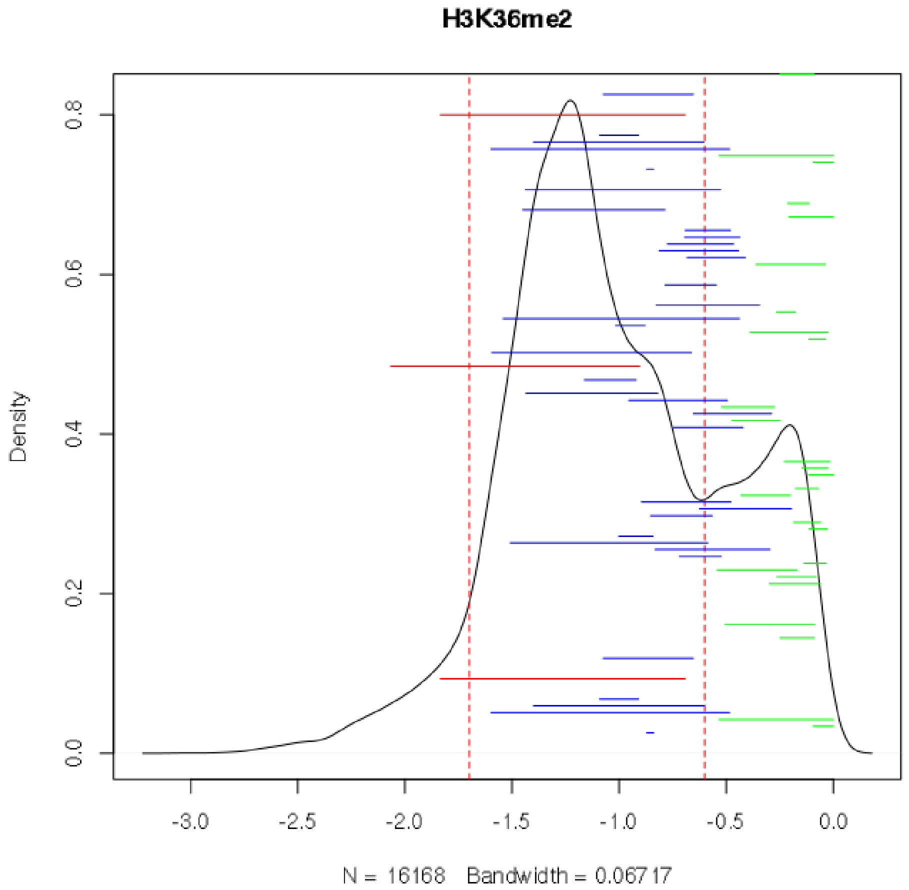

Figure 18: Density plot of the logarithm (base 10) of the proportion of H3K36me2 reads to the total number of H3K36me2 and H3 sequenced reads at each locus. Thresholds for binning the loci are indicated by the red dashed lines. Also shown are horizontal segments describing the confidence intervals on the proportion of H3K36me2 reads for a small number of loci, colour-coded by the bin to which they are allocated; red indicates low H3K36me2 presence, blue moderate H3K36me2 presence, and green high H3K36me2 presence.

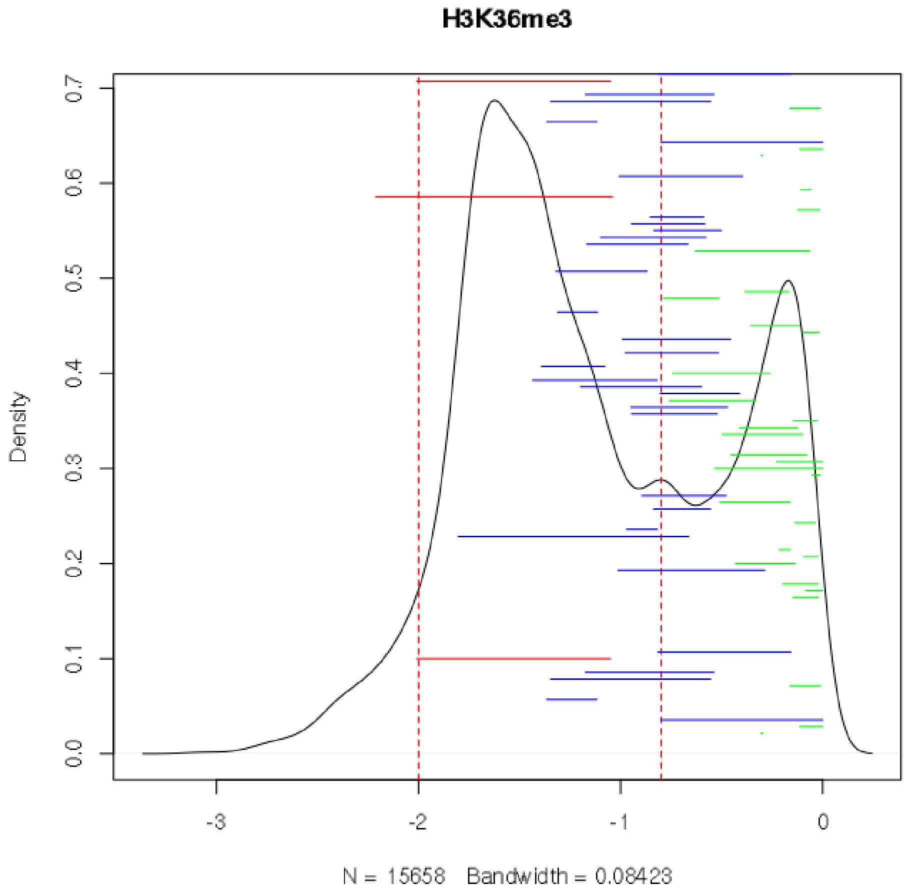

Figure 19: Density plot of the logarithm (base 10) of the proportion of H3K36me3 reads to the total number of H3K36me3 and H3 sequenced reads at each locus. Thresholds for binning the loci are indicated by the red dashed lines. Also shown are horizontal segments describing the confidence intervals on the proportion of H3K36me3 reads for a small number of loci, colour-coded by the bin to which they are allocated; red indicates low H3K36me3 presence, blue moderate H3K36me3 presence, and green high H3K36me3 presence.

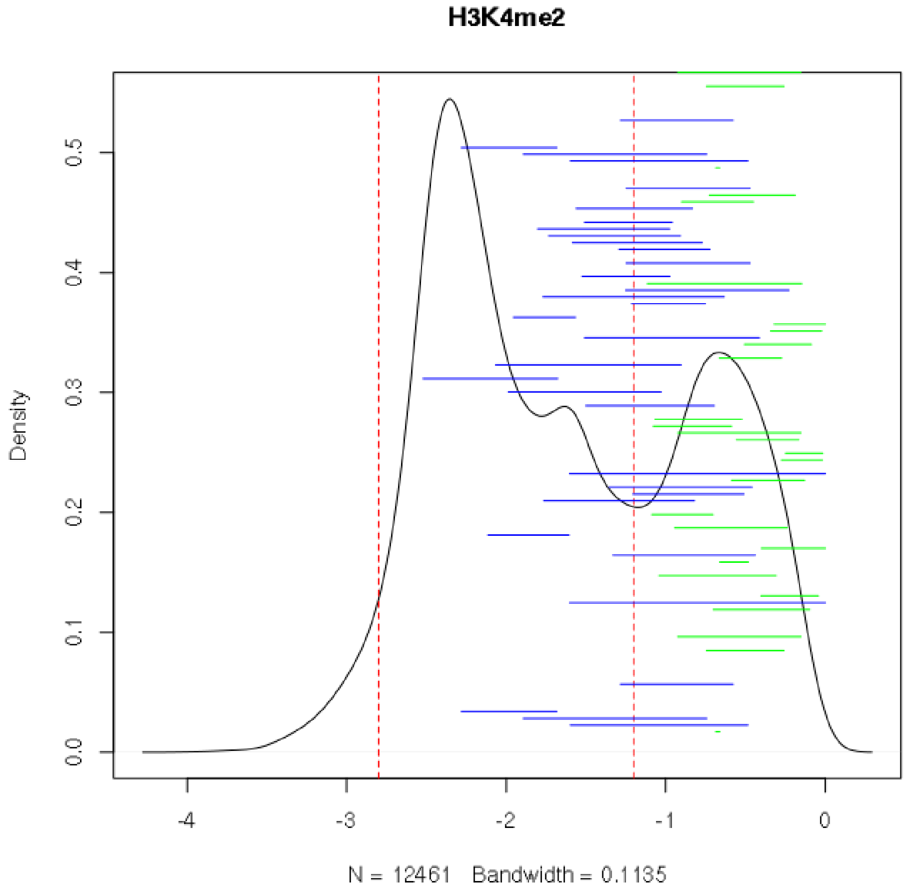

Figure 20: Density plot of the logarithm (base 10) of the proportion of H3K4me2 reads to the total number of H3K4me2 and H3 sequenced reads at each locus. Thresholds for binning the loci are indicated by the red dashed lines. Also shown are horizontal segments describing the confidence intervals on the proportion of H3K4me2 reads for a small number of loci, colour-coded by the bin to which they are allocated; red indicates low H3K4me2 presence, blue moderate H3K4me2 presence, and green high H3K4me2 presence.

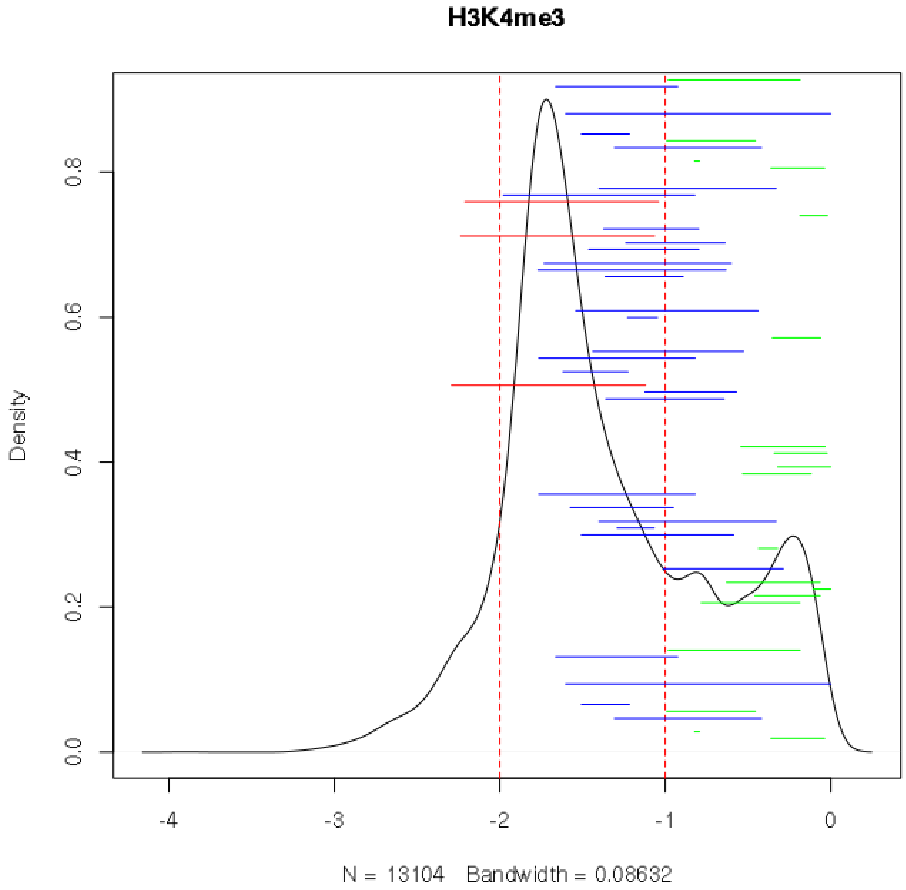

Figure 21: Density plot of the logarithm (base 10) of the proportion of H3K4me3 reads to the total number of H3K4me3 and H3 sequenced reads at each locus. Thresholds for binning the loci are indicated by the red dashed lines. Also shown are horizontal segments describing the confidence intervals on the proportion of H3K4me3 reads for a small number of loci, colour-coded by the bin to which they are allocated; red indicates low H3K4me3 presence, blue moderate H3K4me3 presence, and green high H3K4me3 presence.

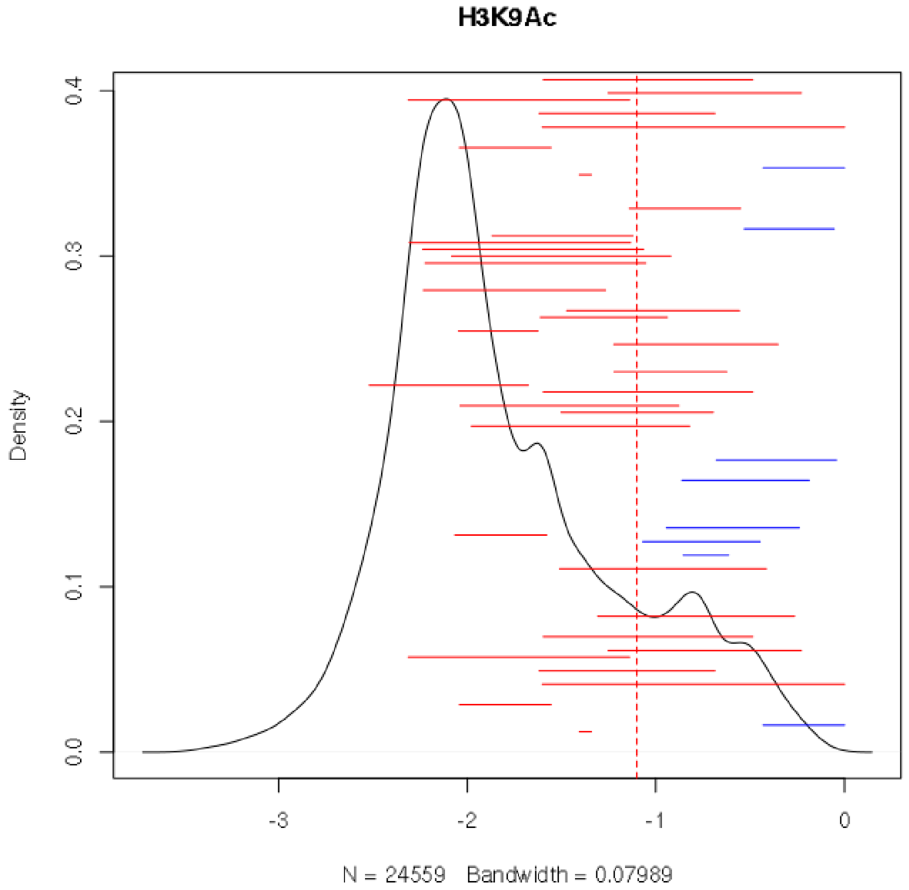

Figure 22: Density plot of the logarithm (base 10) of the proportion of H3K9Ac reads to the total number of H3K9Ac and H3 sequenced reads at each locus. Thresholds for binning the loci are indicated by the red dashed lines. Also shown are horizontal segments describing the confidence intervals on the proportion of H3K9Ac reads for a small number of loci, colour-coded by the bin to which they are allocated; red indicates low H3K9Ac presence and blue high H3K4me2 presence.

References

Hernan Garcia-Ruiz, Atsushi Takeda, Elisabeth J. Chapman, Christopher M. Sullivan, Noah Fahlgren, Katherine J. Brempelis, and James C. Carrington. Arabidopsis RNA-Dependent RNA Polymerases and Dicer-Like Proteins in Antiviral Defense and Small Interfering RNA Biogenesis during Turnip Mosaic Virus Infection. *The Plant Cell Online*, 22 (2):481–496, 2 2010. ISSN 1040-4651, 1532-298X. doi: 10.1105/tpc.109.073056. URL `http://www.plantcell.org/content/22/2/481http://www.ncbi.nlm.nih.gov/pubmed/20190077http://www.plantcell.org/content/22/2/481.full.pdfhttp://www.plantcell.org/content/22/2/481.long`.

Thomas J Hardcastle, Krystyna a Kelly, and David C Baulcombe. Identifying small interfering RNA loci from high-throughput sequencing data. *Bioinformatics (Oxford, England)*, 28(4):457–63, 2 2012. ISSN 1367-4811. doi: 10.1093/bioinformatics/btr687. URL `http://www.ncbi.nlm.nih.gov/pubmed/22171331`.

Ericka R Havecker, Laura M Wallbridge, Thomas J Hardcastle, Maxwell S Bush, Krystyna a Kelly, Ruth M Dunn, Frank Schwach, John H Doonan, and David C Baulcombe. The Arabidopsis RNA-directed DNA methylation argonautes functionally diverge based on their expression and interaction with target loci. *The Plant cell*, 22(2):321–334, 2010. ISSN 1040-4651. doi: 10.1105/tpc.109.072199.

Ericka R. Havecker, Laura M. Wallbridge, Paola Fedito, Thomas J. Hardcastle, and David C. Baulcombe. Metastable Differentially Methylated Regions within Arabidopsis Inbred Populations Are Associated with Modified Expression of Non-Coding Transcripts. *PLoS ONE*, 7(9), 2012. ISSN 19326203. doi: 10.1371/journal.pone.0045242.

Tzuu-fen Lee, Sai Guna Ranjan Gurazada, Jixian Zhai, Shengben Li, Stacey A. Simon, Marjori A. Matzke, Xuemei Chen, and Blake C. Meyers. RNA polymerase V-dependent small RNAs in Arabidopsis originate from small, intergenic loci including most SINE repeats. *Epigenetics : official journal of the DNA Methylation Society*, 7(7):781–95, 7 2012. ISSN 1559-2308. doi: 10.4161/epi.20290. URL `http://www.landesbioscience.com/journals/epigenetics/article/20290/http://www.pubmedcentral.nih.gov/articlerender.fcgi?artid=3679228&tool=pmcentrez&rendertype=abstract`.

Jun Liu, Choonkyun Jung, Jun Xu, Huan Wang, Shulin Deng, Lucia Bernad, Catalina Arenas-Huertero, and Nam-Hai Chua. Genome-Wide Analysis Uncovers Regulation of Long Intergenic Noncoding RNAs in Arabidopsis. *The Plant Cell Online*, 24(11):4333–4345, 11 2012. ISSN 1040-4651, 1532-298X. doi: 10.1105/tpc.112.102855. URL `http://www.plantcell.org/content/24/11/4333http://www.plantcell.org/content/24/11/4333.full.pdfhttp://www.plantcell.org/content/24/11/4333.long`.

Attila Molnar, Charles W Melnyk, Andrew Bassett, Thomas J Hardcastle, Ruth Dunn, and David C Baulcombe. Small silencing RNAs in plants are mobile and direct epigenetic modification in recipient

cells. *Science (New York, N.Y.)*, 328(5980):872–875, 2010. ISSN 0036-8075. doi: 10.1126/science.1187959.

Taiowa A. Montgomery, Seong Jeon Yoo, Noah Fahlgren, Sunny D. Gilbert, Miya D. Howell, Christopher M. Sullivan, Amanda Alexander, Goretti Nguyen, Edwards Allen, Ji Hoon Ahn, and James C. Carrington. AGO1-miR173 complex initiates phased siRNA formation in plants. *Proceedings of the National Academy of Sciences*, 105 (51):20055–20062, 12 2008. ISSN 0027-8424, 1091-6490. doi: 10.1073/pnas.0810241105. URL http://www.pnas.org/content/105/51/20055http://www.ncbi.nlm.nih.gov/pubmed/19066226http://www.pnas.org/content/105/51/20055.full.pdfhttp://www.pnas.org/content/105/51/20055.long.

Rebecca a Mosher, Frank Schwach, David Studholme, and David C Baulcombe. PolIVb influences RNA-directed DNA methylation independently of its role in siRNA biogenesis. *Proceedings of the National Academy of Sciences of the United States of America*, 105(8):3145–3150, 2008. ISSN 0027-8424. doi: 10.1073/pnas.0709632105.

Rebecca a Mosher, Charles W Melnyk, Krystyna a Kelly, Ruth M Dunn, David J Studholme, and David C Baulcombe. Uniparental expression of PolIV-dependent siRNAs in developing endosperm of Arabidopsis. *Nature*, 460(7252):283–286, 2009. ISSN 0028-0836. doi: 10.1038/nature08084. URL http://dx.doi.org/10.1038/nature08084.

Hume Stroud, Truman Do, Jiamu Du, Xuehua Zhong, Suhua Feng, Lianna Johnson, Dinshaw J Patel, and Steven E Jacobsen. Non-CG methylation patterns shape the epigenetic landscape in Arabidopsis. *Nature structural & molecular biology*, 21(1):64–72, 1 2014. ISSN 1545-9985. doi: 10.1038/nsmb.2735. URL http://www.ncbi.nlm.nih.gov/pubmed/24336224.

Robert Tibshirani, Guenther Walther, and Trevor Hastie. Estimating the number of clusters in a data set via the gap statistic. *Journal of the Royal Statistical Society: Series B (Statistical Methodology)*, 63(2):411–423, 2001. ISSN 1369-7412. doi: 10.1111/1467-9868.00293. URL http://doi.wiley.com/10.1111/1467-9868.00293.

Xiaoming Zhang, Jing Xia, Yifan E. Lii, Blanca E. Barrera-Figueroa, Xuefeng Zhou, Shang Gao, Lu Lu, Dongdong Niu, Zheng Chen, Christy Leung, Timothy Wong, Huiming Zhang, Jianhua Guo, Yi Li, Renyi Liu, Wanqi Liang, Jian-Kang Zhu, Weixiong Zhang, and Hailing Jin. Genome-wide analysis of plant nat-siRNAs reveals insights into their distribution, biogenesis and function. *Genome Biology*, 13(3):R20, 3 2012. ISSN 1465-6906. doi: 10.1186/gb-2012-13-3-r20. URL http://genomebiology.com/2012/13/3/R20/abstracthttp://genomebiology.com/2012/13/3/r20http://genomebiology.com/content/pdf/gb-2012-13-3-r20.pdf.
